# Postnatal prolongation of mammalian nephrogenesis by excess fetal GDNF

**DOI:** 10.1242/dev.197475

**Published:** 2021-05-25

**Authors:** Hao Li, Kristen Kurtzeborn, Jussi Kupari, Yujuan Gui, Edward Siefker, Benson Lu, Kärt Mätlik, Soophie Olfat, Ana R. Montaño-Rodríguez, Sung-Ho Huh, Franklin Costantini, Jaan-Olle Andressoo, Satu Kuure

**Affiliations:** 1 Stem Cells and Metabolism Research Program, Faculty of Medicine, University of Helsinki, 00014 Helsinki, Finland; 2 Helsinki Institute of Life Science, University of Helsinki, 00014 Helsinki, Finland; 3 Department of Developmental Neuroscience, University of Nebraska Medical Center, Omaha, NE 68105, USA; 4 Department of Genetics and Development, Columbia University Medical Center, New York, NY 10032, USA; 5 Department of Pharmacology, Faculty of Medicine, University of Helsinki, 00014 Helsinki, Finland; 6 Department of Neurobiology, Care Sciences and Society, Karolinska Institutet, 171 77 Stockholm, Sweden; 7 GM-unit, Laboratory Animal Centre, Helsinki Institute of Life Science, University of Helsinki, 00014 Helsinki, Finland

**Keywords:** Differentiation, Kidney, Nephrogenesis, Nephron progenitors, Mouse

## Abstract

Nephron endowment, defined during the fetal period, dictates renal and related cardiovascular health throughout life. We show here that, despite its negative effects on kidney growth, genetic increase of GDNF prolongs the nephrogenic program beyond its normal cessation. Multi-stage mechanistic analysis revealed that excess GDNF maintains nephron progenitors and nephrogenesis through increased expression of its secreted targets and augmented WNT signaling, leading to a two-part effect on nephron progenitor maintenance. Abnormally high GDNF in embryonic kidneys upregulates its known targets but also *Wnt9b* and *Axin2*, with concomitant deceleration of nephron progenitor proliferation. Decline of GDNF levels in postnatal kidneys normalizes the ureteric bud and creates a permissive environment for continuation of the nephrogenic program, as demonstrated by morphologically and molecularly normal postnatal nephron progenitor self-renewal and differentiation. These results establish that excess GDNF has a bi-phasic effect on nephron progenitors in mice, which can faithfully respond to GDNF dosage manipulation during the fetal and postnatal period. Our results suggest that sensing the signaling activity level is an important mechanism through which GDNF and other molecules contribute to nephron progenitor lifespan specification.

## Abstract (precis)

**Summary:** Dosage of neurotropic factor GDNF regulates nephron progenitors and *in utero* growth factor augmentation can extend postnatal lifespan and differentiation of nephron progenitors.

## INTRODUCTION

The mammalian kidney is a non-regenerating blood filtrating organ with essential detoxification and electrolyte balancing functions. The nephrons executing these functions are born during the fetal period, as the adult kidney shows only limited repair capacity or compensatory glomerular enlargement (hypertrophy) (Chawla and Kimmel, 2012; Rinkevich et al., 2014; Romagnani et al., 2013). Congenital low nephron mass is a widely accepted risk factor for hypertension, proteinuria, glomerulosclerosis and end-stage renal disease (Bertram et al., 2011; Hoy et al., 2008) and thus greatly influences renal health throughout life.

The nephrogenic program ends between gestation weeks 35-37 in humans and postnatal day (P) 3 in mouse (Hartman et al., 2007; Hinchliffe et al., 1991; Ryan et al., 2018), but the mechanisms contributing to the cessation are poorly understood. It has been postulated that stage-specific changes in growth patterns (Cebrián et al., 2004) and cell intrinsic age-sensing mechanisms (Chen et al., 2015a) are involved. Molecularly, bone morphogenetic protein (BMP)-induced SMAD signaling (Brown et al., 2015), hamartin (*Tsc1*) (Volovelsky et al., 2018) and microRNA regulation (Yermalovich et al., 2019) participate in determination of the nephrogenic program duration. However, plasticity of the nephrogenic program has not been fully explored.

Nephrons differentiate from a nephron progenitor (NP) pool, also known as metanephric or cap mesenchyme. NPs expressing a unique repertoire of markers including SIX2, glial cell line derived neurotrophic factor (GDNF) and CITED1, are present only in the embryonic kidney where they surround each ureteric bud (UB) tip from its establishment (Boyle et al., 2008; Self et al., 2006). The nephrogenesis process is synchronized with the UB branching, which simultaneously maintains self-renewal in the undifferentiated NP population and induces a subset of NPs to undergo gradual mesenchyme-to-epithelium transition via well-defined precursor stages (pretubular aggregates, PA; renal vesicles, RV; comma-shaped bodies, CSB; S-shaped bodies, SSB), which eventually differentiate into functional nephrons (Kobayashi et al., 2008; Lindström et al., 2018).

The balance in self-renewal versus differentiation within the NP population depends on signals mediating the reciprocal inductive tissue interactions taking place between the UB and the neighboring mesenchyme. Metanephric mesenchyme and NP-derived signals, such as GDNF and fibroblast growth factors (FGFs), regulate UB morphogenesis, through which the embryonic kidney grows in size, achieves its shape and establishes the collecting duct system (Costantini and Kopan, 2010). GDNF-activated RET signaling in the UB tips regulates a transcriptional profile responsible for the control of collecting duct progenitors and UB morphogenesis (Kurtzeborn et al., 2018; Li et al., 2019; Lu et al., 2009; Ola et al., 2011; Riccio et al., 2016). GDNF-regulated transcripts also comprise secreted proteins, such as WNT11 and CRLF1, with demonstrated potential to regulate the nephrogenic program (O'Brien et al., 2018; Schmidt-Ott et al., 2005). Other UB-derived nephrogenesis regulators are WNT9b and FGF-ligands influencing NP maintenance and differentiation (Barak et al., 2012; Carroll et al., 2005; Kiefer et al., 2012).

We have previously reported that genetic disruption of the *Gdnf* 3′ untranslated region causes a 3- to 6-fold increased expression of endogenous GDNF and results in renal hypoplasia due to the UB branching defect (Kumar et al., 2015). Unlike newborn lethality in the *Gdnf* knockouts, the *Gdnf*^hyper/hyper^ mice survive up to 3 weeks and have revealed the essential role of GDNF in collecting duct progenitor self-renewal (Costantini, 2012; Kumar et al., 2015; Li et al., 2019). Here, we demonstrate that excess GDNF, despite its negative effect on overall kidney growth, can expand NP lifespan, thus further strengthening the newly recognized plasticity of mammalian kidneys.

## RESULTS

### The embryonic nephrogenic program depends on GDNF

**Fig. 1.**
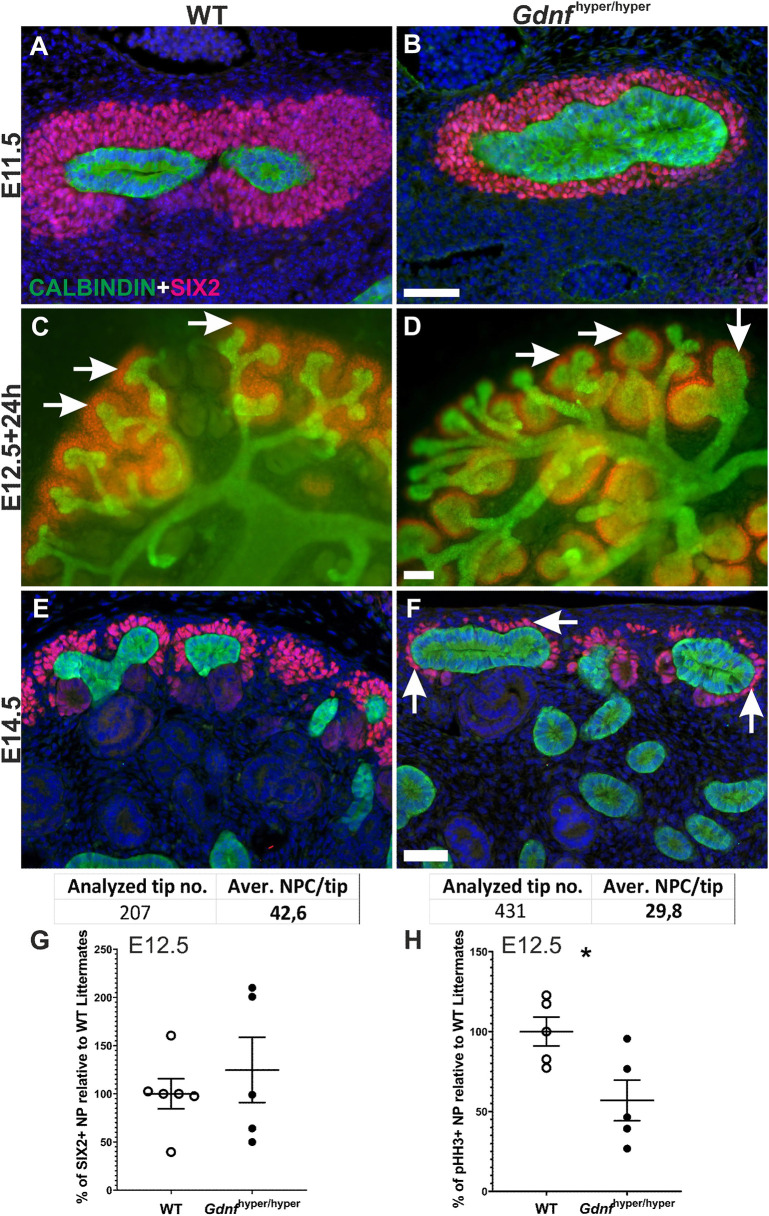
**Nephron progenitors get depleted in embryonic *Gdnf*^hyper/hyper^ kidneys.** (A,B) Nephron progenitor (NP) marker SIX2 (red) localizes to mesenchyme capping ureteric bud (UB) tips (calbindin, green) in WT (A; *n*=4 kidneys) and *Gdnf*^hyper/hyper^ (B; *n*=5 kidneys) kidneys at E11.5. (C,D) E12.5 WT (C) and *Gdnf*^hyper/hyper^ (D) kidneys cultured *in vitro* for 24 h and stained for SIX2 (red) to visualize NP population and calbindin (green) for UB (*n*=10 kidneys/treatment, three independent experiments). (E,F) NPs are abundant in E14.5 WT kidney (E; 42.6/tip), whereas in *Gdnf*^hyper/hyper^ kidney NPs are clearly reduced (F; arrows, 29.8/tip) (*n*=207 for WT and 431 for *Gdnf*^hyper/hyper^ tips analyzed in nine kidneys/genotype). White arrows point to the NP cells (red). (G) Analysis of SIX2-positive NP amount in WT and *Gdnf*^hyper/hyper^ kidneys shows comparable initial NP pools at E12.5. Data are presented as mean quantity of SIX2-positive NPs±s.e.m. compared with those of WT littermates, which are set to 100% and reflect the results obtained from four independent litters (WT: 100±15.61%, *n*=6; *Gdnf*^hyper/hyper^: 124.74±33.89%, *n*=5; *P*=0.533, two-tailed *t*-test in SPSS). (H) Proportion of proliferating SIX2-positive NPs in *Gdnf*^hyper/hyper^ kidneys at E12.5 shows a significant decrease when compared with WT kidney. Data are presented as mean proportion of proliferating SIX2-positive NPs±s.e.m. compared with those of WT littermates which are set to 100% and reflect the results obtained from three independent litters (WT: 100±9.04%, *n*=5; *Gdnf*^hyper/hyper^: 56.94±12.67%, *n*=5; *P=0.024, two-tailed *t*-test in SPSS). Scale bars: 100 µm.

The spatial arrangement of SIX2-positive NPs in the embryonic and early postnatal kidneys follows a stereotypical, well-described pattern, in which the initial multi-cell-layered tissue thins over time without much affecting the overall niche organization (Short et al., 2014). NPs in *Gdnf*^hyper/hyper^ kidneys were distributed around abnormally wide UB as a thinner layer (Fig. 1A-F; Fig. S1A,B). Quantification of NPs at E11.5 revealed an initial increase, which was transiently normalized at E12.5 but severely decreased at E14.5 in *Gdnf*^hyper/hyper^ kidneys (Fig. 1E-G; Fig. S1C-F). Mitosis analysis demonstrated that both endogenously increased and exogenously supplemented GDNF causes significant reduction in pHH3+ NPC (Fig. 1H; Fig. S1G). Collectively, the data show that excess GDNF, which primarily functions in the UB, where its receptors are expressed (Pachnis et al., 1993), induces a rapid drop-off in NP cell proliferation during early kidney development.

**Fig. 2.**
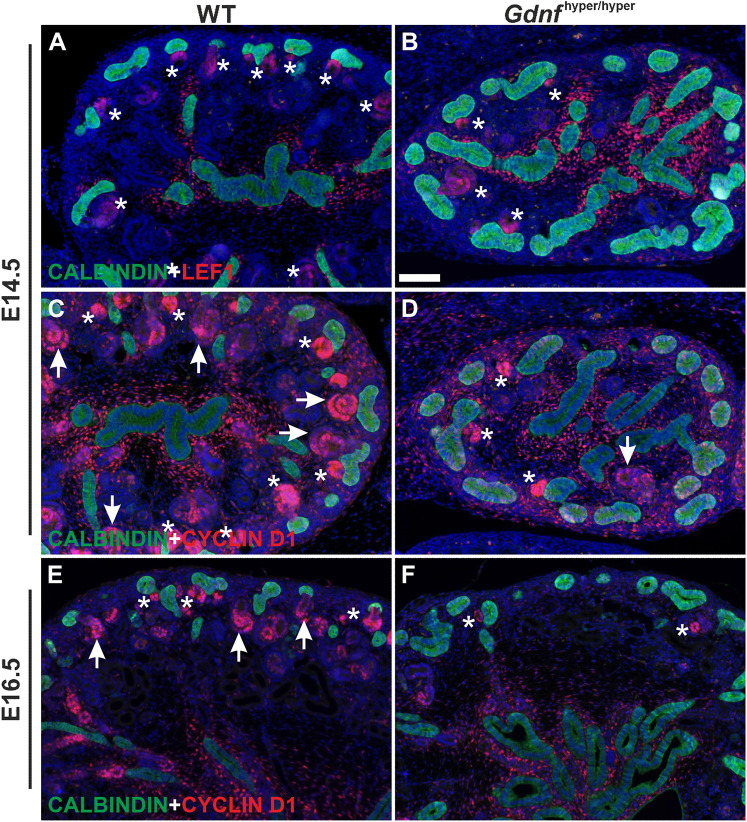
**Effect of excess GDNF on embryonic nephrogenesis.** (A) LEF1 (red), a marker of pretubular aggregates, distal renal vesicles and S-shaped bodies, in WT E14.5 kidney. (B) E14.5 *Gdnf*^hyper/hyper^ kidney shows very few LEF1-positive differentiating nephron precursors and typical abundance of ureteric bud (UB) epithelium as detected by calbindin staining (green). (C) Cyclin D1 (red) stains all precursors of differentiating nephrons (asterisks mark pretubular aggregates and comma-shaped bodies, arrows point to S-shaped bodies), which can be observed abundantly next to UB epithelium (green) in E14.5 WT kidney. (D) Significantly fewer differentiating nephron precursors are detected in E14.5 *Gdnf*^hyper/hyper^ kidney. (E,F) Cyclin D1 (red) localization in E16.5 WT (E) and *Gdnf*^hyper/hyper^ (F) kidneys. Calbindin (green) staining visualizes UB epithelium in all images. For each staining at given stage *n*=3 kidneys/genotype. Scale bar: 100 µm.

It is known that premature or accelerated NP differentiation can cause NP cell decline in their niche (Kobayashi et al., 2008; O'Brien, 2018). To assess this, nephrogenesis was examined by staining with LEF1 and cyclin D1, which label the earliest differentiation-committed cells and nephrons undergoing full epithelialization, respectively (Lindström et al., 2015). This showed a general decline in early nephron precursor numbers and indicated reduced nephron differentiation in embryonic *Gdnf*^hyper/hyper^ kidneys (Fig. 2). Glomerular density assessment further supported this and demonstrated lower overall densities in hypodysplastic *Gdnf*^hyper/hyper^ kidneys than in wild-type (WT) kidneys (Fig. S1G). Interestingly, analysis of the cortical glomeruli within the total measured area, and especially cortical-to-total glomeruli ratios, revealed slightly higher than WT ratios in *Gdnf*^hyper/hyper^ (Table 1; 17% in *Gdnf*^hyper/hyper^, 12% in WT). This indicates that despite the early deceleration of NP self-renewal and differentiation, the kidneys facing excess GDNF during organogenesis have ongoing postnatal nephrogenesis with a minor increase in the latest born nephrons, which are most cortically located (Rumballe et al., 2011; Short et al., 2014).

**Table 1.**
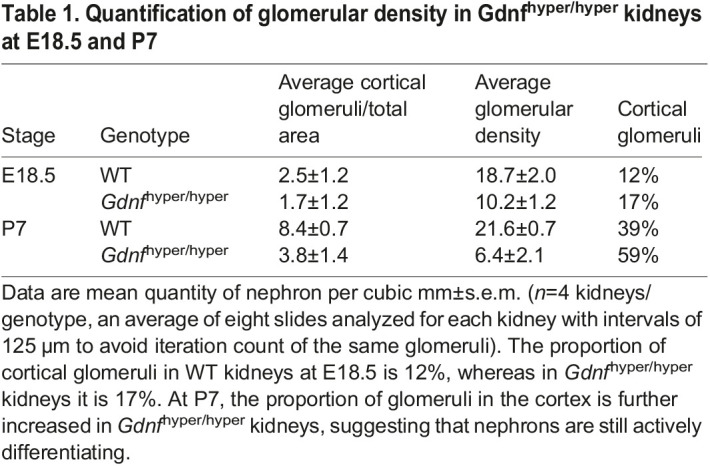
Quantification of glomerular density in Gdnf^hyper/hyper^ kidneys at E18.5 and P7

### Excess GDNF prolongs the lifespan of the postnatal NP pool

Many mouse models with deficiency in kidney growth, either due to UB morphogenesis and/or nephron differentiation, die immediately after birth (Kuure and Sariola, 2020). Despite the significant hypoplasia, abnormalities in UB morphology and severely disrupted embryonic nephrogenesis (Fig. 2), *Gdnf*^hyper/hyper^ mice survive up to 3 weeks after birth (Kumar et al., 2015; Li et al., 2019), providing a possibility to examine postnatal nephrogenesis.

**Fig. 3.**
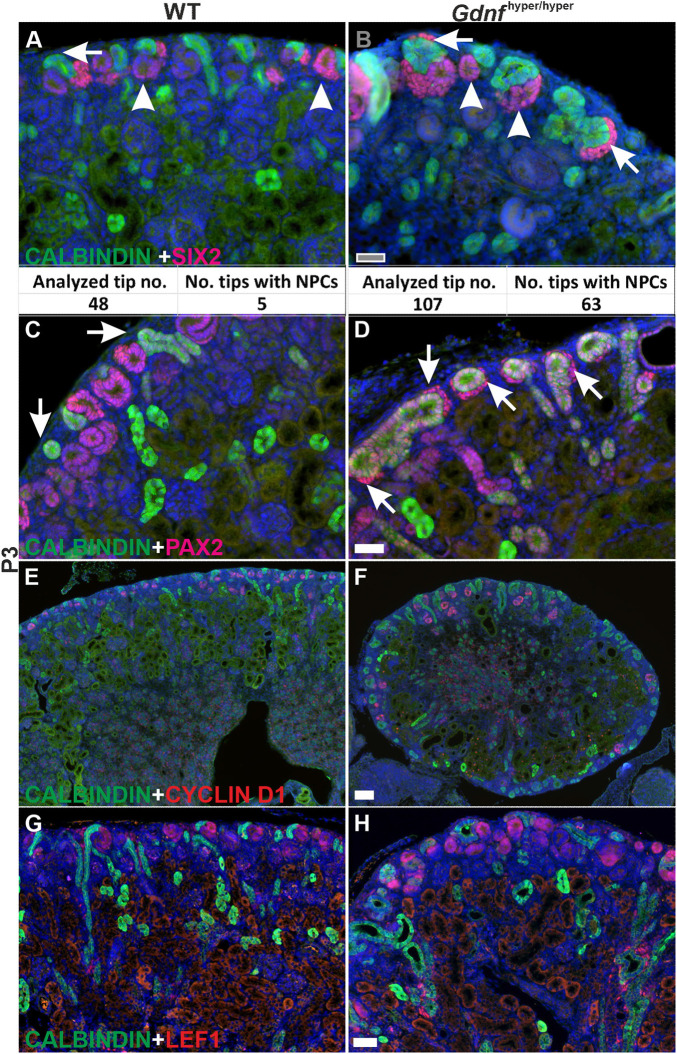
**Nephron differentiation at the cessation of renal morphogenesis.** (A) P3 WT kidney has lost cortical SIX2-positive nephron progenitors (NP) as shown by the loss of cells in the cap mesenchyme (arrow). Instead, SIX2 localizes to lateral mesenchyme and early nephron precursors (arrowheads). Quantification of 48 NP niches (analyzed tip numbers) revealed only five niches with NPs. (B) Cap mesenchyme positive for SIX2 is still present in *Gdnf*^hyper/hyper^ kidney at P3 (arrows). Quantification of 107 NP niches (analyzed tip numbers) revealed 63 niches with NP cells. (C,D) PAX2 (red) and calbindin (green) staining in WT (C) and *Gdnf*^hyper/hyper^ (D) kidneys at P3. Arrows point to the position where NP cells are maintained in *Gdnf*^hyper/hyper^ kidneys. (E,F) Comparable amount of cyclin D1-positive (red) nephron precursors in P3 WT (E) and *Gdnf*^hyper/hyper^ (F) kidneys. (G,H) Visualization of LEF1-positive nephron precursors (red) and ureteric epithelium (green) reveals similar ongoing nephrogenesis in WT (G) and *Gdnf*^hyper/hyper^ (H) kidneys. For each staining *n*=3 kidneys/genotype. Scale bars: 100 µm.

As has been established (O'Brien, 2018), our analysis of the NP populations at P1-P10 revealed dramatic loss in the WT kidneys at P3, in which SIX2-positivity was mainly detected in the differentiating nephron precursors, and only 10% of the analyzed UB tips (niches) were capped with nephron progenitor cells (NPCs) (Fig. 3A, *n*=2 kidneys). However, SIX2-positive NPs were detected in 59% of *Gdnf*^hyper/hyper^ niches at P3 (Fig. 3B, *n*=2 kidneys). Similarly, PAX2-positive NPs were detected in NP niches of *Gdnf*^hyper/hyper^ kidneys at P3, but the PAX2-positive cells localized exclusively in the differentiating nephron precursors in WT kidneys (Fig. 3C,D).

**Fig. 4.**
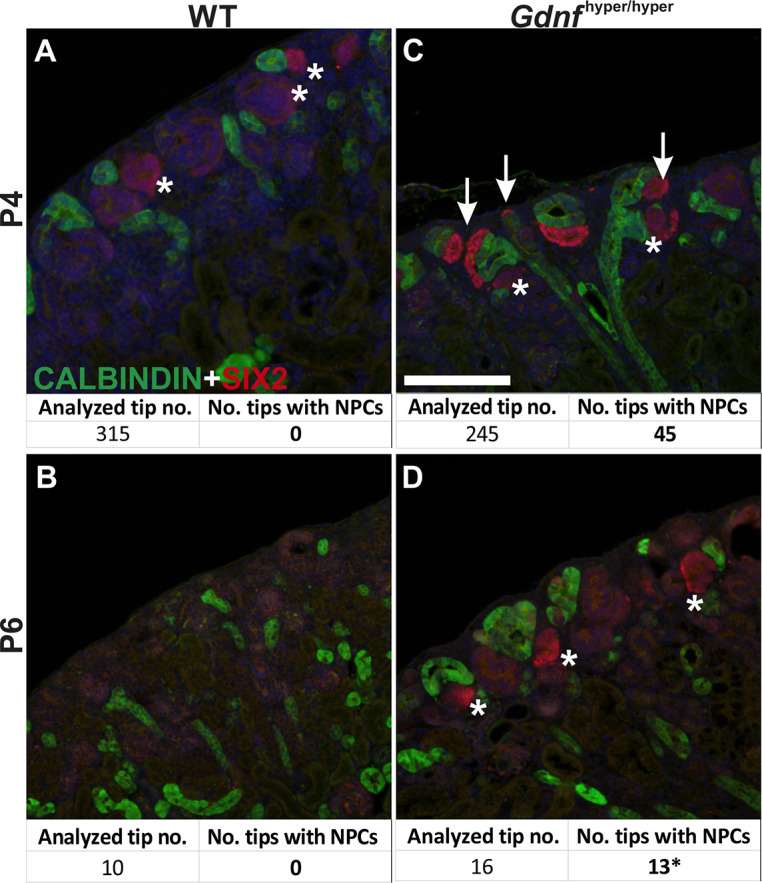
**Nephron progenitors are persistently sustained in the postnatal *Gdnf*^hyper/hyper^ kidneys.** (A,B) Localization of nephron progenitor (NP) marker SIX2 (red) in WT at P4 (A) and P6 (B) shows loss of NPs in WT kidneys in which SIX2 is found only in weakly positive renal vesicles (asterisks). (C) In *Gdnf*^hyper/hyper^ kidneys at P4 SIX2 localizes also to NPs capping the ureteric bud (green) tips (arrows) (*n*=315 tips in WT and 245 in *Gdnf*^hyper/hyper^ analyzed in four kidneys/genotype). (D) Localization of SIX2 (red) and calbindin (green) in P6 *Gdnf*^hyper/hyper^ kidneys reveals the persistence of committed NP cells (13; * indicates differentiating nephrons) in the mutant kidney (*n*=3 kidneys/genotype). Quantification of NP cell niches showed very few tips, which did not have any SIX2-positive NPs in WT kidneys, whereas 81% of niches in *Gdnf*^hyper/hyper^ kidneys (*n*=2, 16 niches) were accompanied with SIX2-positivity in committed and differentiating nephron precursors. Scale bar: 100 µm.

Nephrogenesis in postnatal *Gdnf*^hyper/hyper^ kidneys demonstrated significant improvement from that in embryonic kidneys, as exemplified by similar nephron precursor patterns in WT and *Gdnf*^hyper/hyper^ kidneys (Fig. 3E-H; compare with Fig. 2). Finally, we found that ∼18% of the NP niches sustained SIX2-positive NPCs at P4, and committed NPCs were present in *Gdnf*^hyper/hyper^ kidneys until P6, whereas committed NPCs were detected in WT kidneys only until P4 (Fig. 4; Fig. S1H). This demonstrates that the NP lifespan *in vivo* is not pre-fixed, and suggests that modulation of growth factor levels, specifically GDNF, contributes to the timing of the final nephron differentiation wave.

We hypothesized that persistent NPCs in postnatal *Gdnf*^hyper/hyper^ kidneys could either result from general compensatory mechanisms provoked by UB branching defects leading to renal hypoplasia (Kumar et al., 2015; Li et al., 2019) or alternatively derive from the GDNF-specific effects on the NP population. To discriminate between the options, postnatal NPs were analyzed in other models of renal hypoplasia. *Fgf9;20*-deficient kidneys show similar thinning of embryonic NP cell layers (Barak et al., 2012) as detected in *Gdnf*^hyper/hyper^ kidneys, but we failed to detect SIX2- and/or PAX2-positive cells in their postnatal NP niches (Fig. S2). To further elaborate on this, we next analyzed *Hoxb7Cre; Mek1^fl/fl^;Mek2^+/−^* kidneys (Ihermann-Hella et al., 2014) in which, similar to *Gdnf^hyper/hyper^* mice, renal hypoplasia is caused by impaired UB branching. This model also lacked signs of prolonged nephrogenesis, as detected by the presence of SIX2- and/or PAX2-positive cells in their postnatal NP niches (Fig. S3), supporting the view that renal hypoplasia as such is not enough to maintain postnatal nephrogenesis.

Published data also demonstrates that renal hypoplasia alone is unable to support postnatal NP maintenance (Kuure and Sariola, 2020), which further suggests an active role for GDNF. However, we cannot completely exclude the possibility that excess growth factors other than GDNF would have the same effect.

### Sustained postnatal NPs maintain the nephrogenic program beyond its normal cessation

**Fig. 5.**
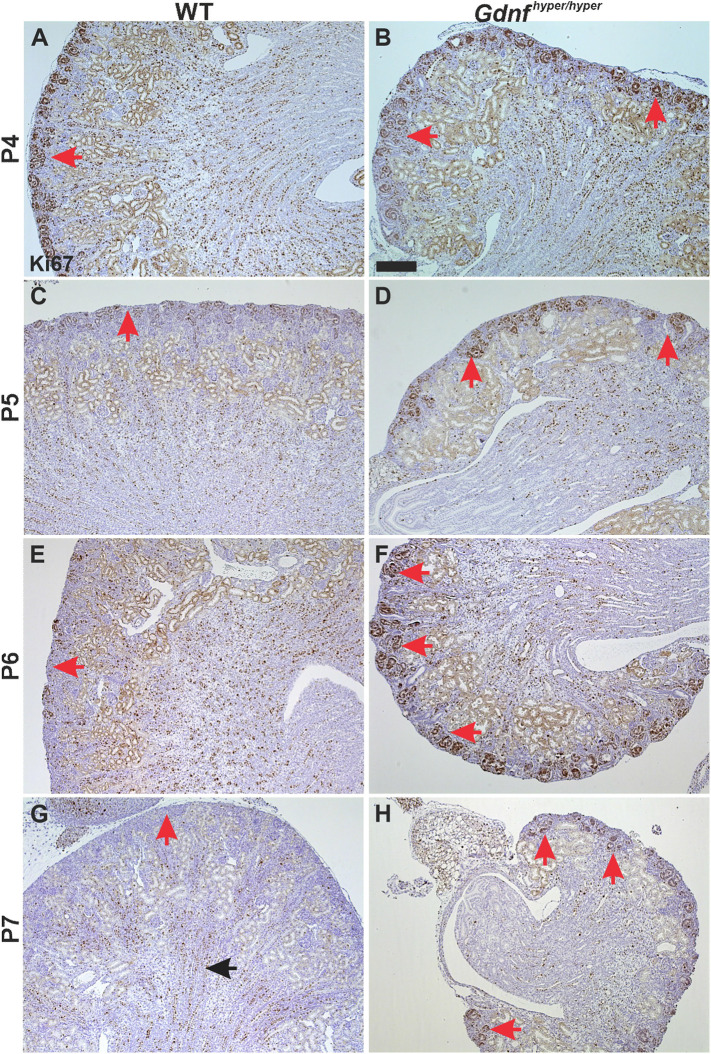
**Sustained cortical proliferation in postnatal *Gdnf*^hyper/hyper^ kidneys.** (A,B) Ki67 staining in P4 WT (A) and *Gdnf*^hyper/hyper^ (B) kidneys shows similar pattern of proliferative cells in the cortical kidneys (red arrows) of both genotypes. (C,D) In P5 WT kidneys (C) Ki67-positivity is significantly reduced in the cortex (red arrow), whereas *Gdnf*^hyper/hyper^ kidneys (D) still exhibit focally very high proliferation (red arrows). (E,F) Proliferation in the WT cortical kidney (E) is further diminished at P6, whereas it remains highly active in *Gdnf*^hyper/hyper^ kidneys (F). (G,H) Ki67-positive proliferative cells localize to medullar renal tubules (black arrow) in P7 WT kidneys (G) but active proliferation is still detected in cortical differentiating nephron structures (red arrows) of *Gdnf*^hyper/hyper^ kidneys (H). For each postnatal stage, *n*=3 kidneys/genotype. Scale bar: 1 mm.

Next, the differentiation potential of sustained NPs in the postnatal *Gdnf*^hyper/hyper^ kidneys was examined. Ki67 analysis revealed comparable postnatal proliferation patterns in WT and *Gdnf*^hyper/hyper^ kidneys until P4 (Fig. 5A,B). From P5 onwards, the Ki67-positive cells decreased in the cortical differentiation zone of WT kidneys (Fig. 5C,E,G). Such a drop in cortical proliferation was not detected in *Gdnf*^hyper/hyper^ kidneys, which showed actively cycling cells in nephron precursor-like structures at P7 (Fig. 5D,F,H) but no longer at P12 (Fig. S4A,B).

**Fig. 6.**
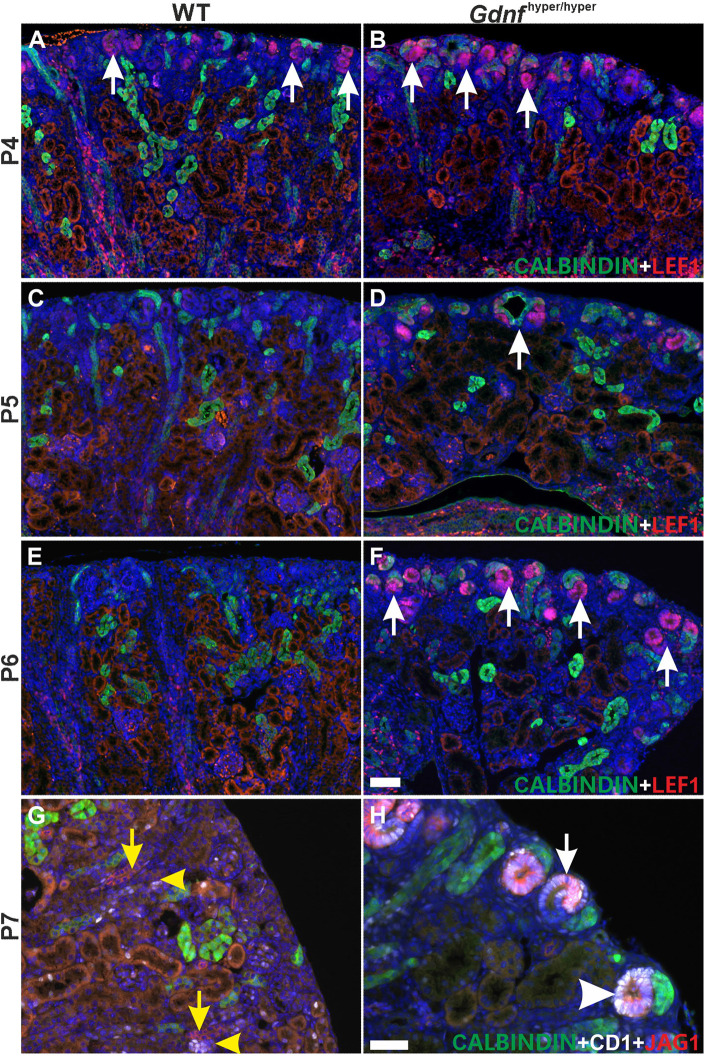
**Formation of new nephrons occurs until P7 only in *Gdnf*^hyper/hyper^ kidneys.** (A,B) Nephron precursor marker LEF1 (red) in WT (A) and *Gdnf*^hyper/hyper^ (B) kidneys at P4 demonstrates ongoing nephrogenesis in both genotypes (arrows) (*n*=2 kidneys/genotype). (C,D) P5 WT kidneys (C) show very few LEF1-positive cells in cortex, whereas ample nephrogenesis is detected in *Gdnf*^hyper/hyper^ kidneys (D; arrows; *n*=4 kidneys/genotype). (E,F) LEF1 is no longer present in the cortex of WT kidneys at P6 (E), whereas plentiful nephron precursors are still detected in *Gdnf*^hyper/hyper^ kidneys (F) (*n*=3 kidneys/genotype). (G) Cyclin D1 (white) and JAG1 (red) localize to differentiated distal tubules of nephron (yellow arrow and arrowhead) in P7 WT kidney (*n*=3 kidneys). (H) In P7 *Gdnf*^hyper/hyper^ kidneys cyclin D1 and JAG1 localize to comma-shaped (arrowhead) and S-shaped (arrow) bodies of nephron precursors in the cortical differentiation zone, thus showing sustained nephrogenesis at this late postnatal stage (*n*=3 kidneys). Scale bars: 100 µm.

Postnatal nephron differentiation analysis demonstrated an excessive quantity of nephron precursors in *Gdnf*^hyper/hyper^ kidneys until P7, whereas these were detected in WT kidneys only up to P4 (Fig. 6; Fig. S4C-F). Such persistent postnatal nephrogenesis, however, failed to increase glomerular density from that in late embryonic stages (Fig. S5A-C; 6.4±2.1 versus 10.2±1.2, respectively; mean±s.e.m.). The cortical glomeruli proportion in *Gdnf*^hyper/hyper^ kidneys at P7 (59%) is 1.5× higher than in WT kidneys (39%) (Table 1) supporting the view that the sustained postnatal NP pool in *Gdnf*^hyper/hyper^ kidneys induces new nephrons after the normal nephrogenesis cessation.

*Gdnf*^hyper/hyper^ mice with prolonged nephrogenesis in severely hypodysplastic kidneys show no signs of neoplasia or tumorigenesis during their short lifespan (Kumar et al., 2015; Turconi et al., 2020). *Gdnf*^wt/hyper^ kidneys show milder embryonic decline of NPCs than *Gdnf*^hyper/hyper^ kidneys. These mice live a normal lifespan and do not show tumors in kidneys and other organs (Turconi et al., 2020) (Fig. S6A-D, *n*=62). The twofold GDNF increase during embryogenesis, however, fails to maintain the NP population in postnatal *Gdnf*^wt/hyper^ kidneys (Fig. S6E,F), suggesting a tight threshold-limit for the ability of GDNF to modulate the nephrogenic program.

### Persistent postnatal nephrogenesis depends on changes in GDNF-induced signaling

**Fig. 7.**
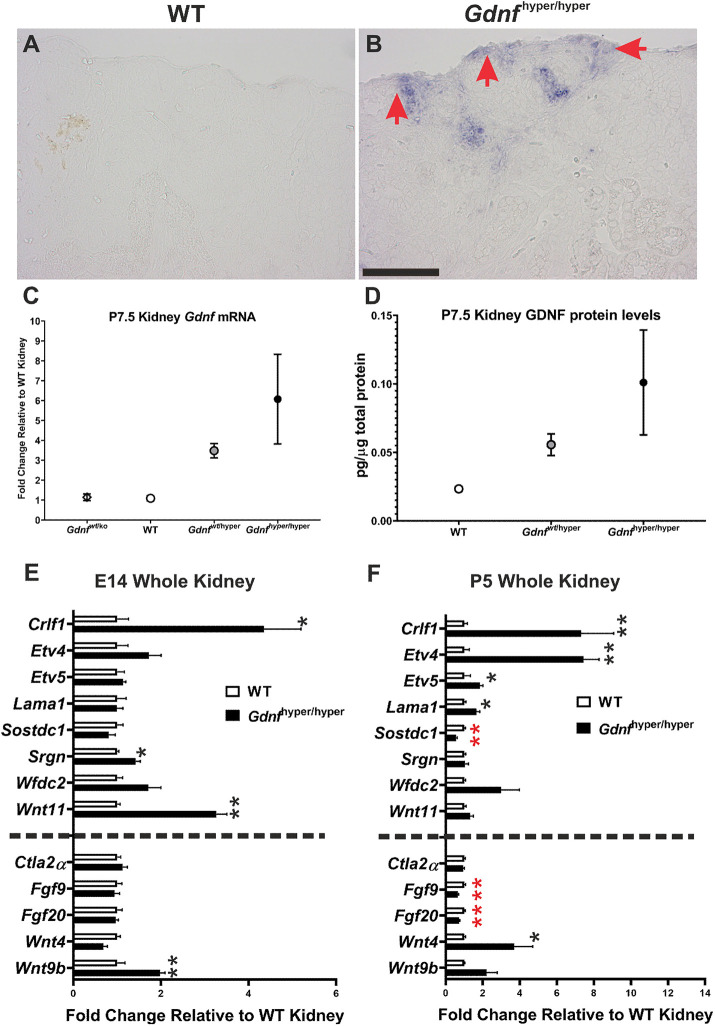
**Expression analysis of the crucial renal development regulators.** (A) *In situ* hybridization assay of *Gdnf* at P4 is unable to detect any transcripts in WT kidney (*n*=3 kidneys). (B) *Gdnf* expression is maintained in P4 *Gdnf*^hyper/hyper^ kidney and localizes to cortical differentiation zone in which nephron progenitors are maintained (red arrows) owing to excess GDNF (*n*=3 kidneys). (C) qRT-PCR analysis of *Gdnf* transcripts in *Gdnf*^wt/ko^, WT, *Gdnf*^wt/hyper^ and *Gdnf*^hyper/hyper^ kidneys at P7 (*n*=3 kidneys/genotype). *Gdnf* expression in *Gdnf*^hyper/hyper^ kidneys is significantly higher than in WT (*P*=0.036) and *Gdnf*^wt/ko^ (*P*=0.038) (mean±s.e.m., two-tailed *t*-test in SPSS). (D) ELISA analysis of GDNF protein levels in P7 kidneys (*n*=5 kidneys/genotype) (mean±s.e.m.). (E,F) qRT-PCR based quantification of the relative expression levels of the selected genes at E14 (E) and at P5 (F) (*n*=3 kidneys/genotype at given stage). The UB-derived secreted GDNF targets are listed above the dashed line, whereas other known nephrogenesis regulators are presented below the line. *Gdnf*^hyper/hyper^ kidneys show significant upregulation of *Crlf1* (*P=0.019), *Srgn* (*P=0.021), *Wnt11* (**P=0.001) and *Wnt9b* (**P=0.01) at E14, whereas at P5, significantly increased gene expressions include *Crlf1* (**P=0.009), *Etv4* (**P=0.000), *Etv5* (*P=0.03), *Lama1* (*P=0.014) and *Wnt4* (*P=0.026). In contrast, the transcription of *Sostdc1* (**P=0.000), *Fgf9* (**P=0.001) and *Fgf20* (**P=0.001) is significantly decreased in *Gdnf*^hyper/hyper^ kidneys at P5. Scale bar: 100 µm.

*Gdnf* expression is lost from WT kidneys by P2 (www.gudmap.org), whereas its mRNA and protein are still present in *Gdnf*^hyper/hyper^ kidneys as late as P7 (Fig. 7A-D). The conservative GDNF receptor complex is expressed only in the epithelial UB tips (Costantini, 2012; Golden et al., 1999). Owing to the receptor localization in the tissue adjacent to the NPC population, we suspected that the effects of increased endogenous GDNF on nephrogenesis must be mediated through UB-derived, secreted, GDNF-dependent molecule(s) (http://www.signalpeptide.de/index.php) (Lu et al., 2009; Ola et al., 2011; Schmidt-Ott et al., 2005) (Table 2), or other molecules, the expression of which could change due to the oversized UB.

**Table 2.**
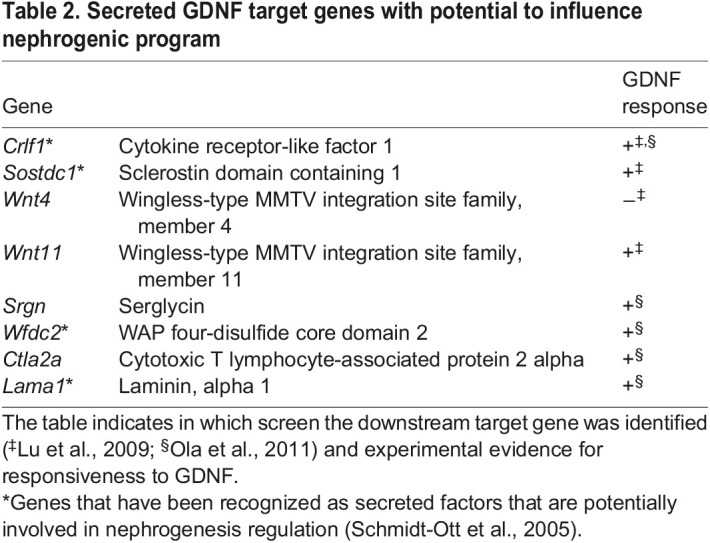
Secreted GDNF target genes with potential to influence nephrogenic program

The expression analysis of UB-derived, secreted GDNF-targets revealed significant upregulation of *Crlf1*, *Srgn* and *Wnt11* in *Gdnf*^hyper/hyper^ kidneys at E14.5 (Fig. 7E). Also, *Wnt9b* expression is significantly increased (Fig. 7E). Further analysis of the WNT/β-catenin signaling targets (Clevers and Nusse, 2012) in E14.5 control and *Gdnf*^hyper/hyper^ kidneys demonstrated significant upregulation of canonical target *Axin2* (*P*=0.003371), whereas NP-expressed WNT targets (Karner et al., 2011) showed a trend of downregulation, likely reflecting the reduced overall number of NPCs (Fig. S7A). Together, these indicate that not only augmented GDNF-induced signaling but also increased *Wnt9*- and *Wnt11*-induced signaling with known functions in NPC regulation participate in mediating reciprocal communication from mutant UB to the adjacent NP pool at E14.5 (Karner et al., 2011; Kiefer et al., 2012; Nagy et al., 2016; O'Brien et al., 2018).

Molecular characterization of P5 *Gdnf*^hyper/hyper^ kidneys showed persistent upregulation of *Crlf1*, *Etv4*, *Etv5*, *Cited1* and *Uncx4.1* (also known as *Uncx*), but also revealed increased expression of potential niche factor *Lama1* (Rayagiri et al., 2018), nephron differentiation inducer *Wnt4* (Stark et al., 1994) and WNT signaling agonist R-spondin 1 (*Rspo1*) (Fig. 7F; Fig. S7B). Such a transcriptional profile suggests an augmented UB-derived effect on NPs, which appear to undergo a differentiation wave based on the decreased *Fgf9* and *Fgf20* expression and shifted localization of SIX2+ cells (Figs 4C,D and 7F).

We functionally tested whether the most significantly increased *Crfl1*, *Wnt11* and *Wnt9b* expressions possibly affect the nephrogenic program in mouse. CRLF1, in complex with its physiological ligand cardiotrophin-like cytokine, induces differentiation in isolated rat kidney mesenchyme cultures (Schmidt-Ott et al., 2005). Characterization of *Crfl1*-knockout kidneys revealed comparable kidney size and nephrogenesis with WT kidneys (Fig. S6C-F), suggesting dispensable CRLF1 functions *in vivo*.

The contribution of increased canonical WNT signaling to the phenotypes caused by excess GDNF was tested next. IWR1 inhibits tankyrases 1 and 2, which are necessary for normal kidney development (Chen et al., 2009; Huang et al., 2009; Karner et al., 2010). IWR1 treatment alleviated UB tip numbers and morphology in the presence of excess GDNF (Fig. S8). Finally, we asked whether significantly increased *Wnt11* expression in *Gdnf*^hyper/hyper^ kidneys contributes to the prolonged nephrogenic program in *Gdnf*^hyper/hyper^ mice by crossing *Wnt11* knockout-allele to the *Gdnf*^hyper^ background. This failed to generate double homozygote *Gdnf*^hyper/hyper^*;Wnt11*^−/−^ offspring (Table 3; *n*=95; *P*=0.005 *Gdnf*^wt//hyper^*;Wnt11*^+/−^ breeding). These results indicate embryonic lethality for *Gdnf*^hyper/hyper^*;Wnt11^−/−^* mutants and forced us to focus the analysis on *Gdnf*^hyper/hyper^*;Wnt11^+/−^* kidneys.

**Table 3.**
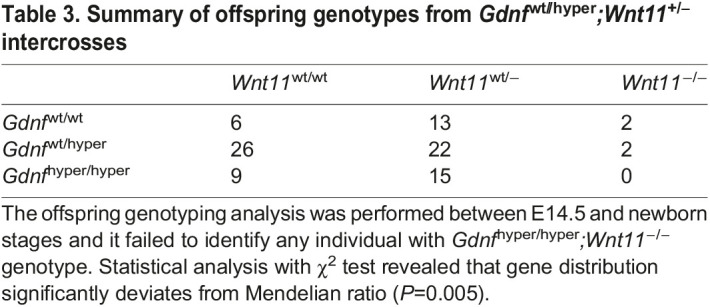
Summary of offspring genotypes from *Gdnf*^wt//hyper^*;Wnt11^+/−^* intercrosses

**Fig. 8.**
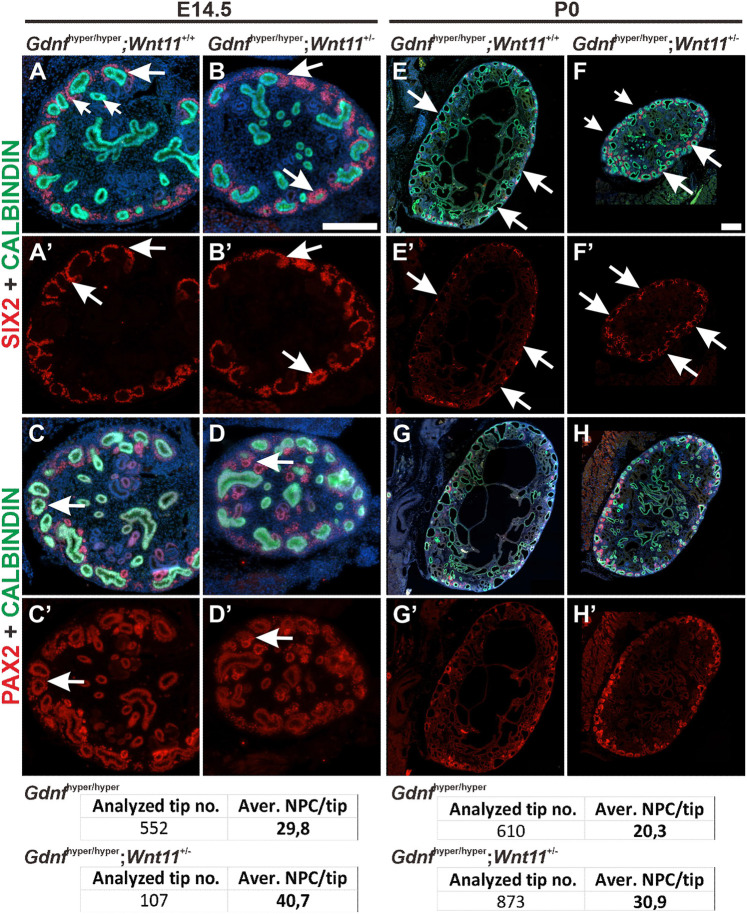
**Genetic decrease of *Wnt11* level partially rescues nephron progenitor loss in *Gdnf*^hyper/hyper^ kidneys.** (A) SIX2-positive nephron progenitors (NP, red) are sparse in E14.5 *Gdnf*^hyper/hyper^ kidneys (average of 29.8 NPs/tip in 552 tips analyzed in nine kidneys). (B) *Gdnf*^hyper/hyper^*;Wnt11*^+/−^ kidneys show an increase in general abundance of NPs per niche in the absence of one *Wnt11* allele (average of 40.7 NPs/tip in 107 tips analyzed in four kidneys). (C,D) PAX2-positive cells in *Gdnf*^hyper/hyper^ (C) and *Gdnf*^hyper/hyper^*;Wnt11*^+/−^ (D) kidneys show similar patterns and amount at E14.5. (E-H) At P0, SIX2 staining demonstrates an improvement in NP numbers by the removal of one *Wnt11* allele (average of 20.3 NPs/tip in 610 *Gdnf*^hyper/hyper^ tips versus average of 30.9 NPs/tip in *Gdnf*^hyper/hyper^*;Wnt11*^+/−^ tips analyzed in six kidneys/genotype), which is supported by PAX2 staining. Distribution of SIX2-positive NPs (A′,B′,E′,F′) and PAX2-positive cells (C′,D′,G′,H′) are shown without calbindin (green) signal, which depicts the UB epithelium in all kidneys. Scale bars: 200 µm.

Removal of one *Wnt11* allele in *Gdnf*^hyper/hyper^ background slightly improved UB morphology and reduced the kidney size further from that in *Gdnf*^hyper/hyper^ kidneys, likely reflecting reduced formation of collecting duct cysts upon *Wnt11* dosage decrease (Fig. S9A-C). However, SIX2-positive NP population was enhanced (29.8 NPC/tip versus 40.7 NPC/tip at E14.5 and 20.3 NPC/tip versus 30.9 at P0) and nephrogenesis was improved in the cortical differentiation zone of *Gdnf*^hyper/hyper^*;Wnt1^+/−^* kidneys (Fig. 8; Fig. S9D-F; compare also with Fig. 1F). Thus, these results suggest that increased GDNF levels augment several bud-derived GDNF/Ret targets, which, not alone but in combination with each other and together with increased canonical WNT signaling, are regulating NPCs in embryonic kidneys.

## DISCUSSION

Nephron count is defined during fetal life and can vary greatly between individuals. NP expansion and lifespan greatly influences the final nephron endowment, and therefore identification of regulatory mechanisms that control NP self-renewal is important.

Here, we show that the neurotropic factor GDNF, known to initiate renal development and regulate UB morphogenesis (Costantini, 2010; Kurtzeborn et al., 2018; Li et al., 2019), is, despite its negative effects on kidney growth, capable of prolonging the nephrogenic program beyond its normal cessation. Our results demonstrate that excess GDNF has a bi-phasic effect on NP self-renewal and maintenance. Based on our analysis, we propose a model of how excess GDNF in *Gdnf*^hyper/hyper^ kidneys functions through mechanisms that involve changes in cell cycle progression and capacity to sense growth factor levels.

Initially in embryonic kidneys, high GDNF levels cause a rapid decline in NP numbers per niche, which is typical for many mouse models with primary deficiency in UB (Carroll and Das, 2013; Chen et al., 2015b; Costantini and Kopan, 2010; Liu et al., 2018). The premature NP decline seen in these previously published models is typically caused by their untimely differentiation, which is not the case in *Gdnf*^hyper/hyper^ kidneys. Instead, excess GDNF is capable of maintaining active cell cycle in the postnatal kidney cortex significantly longer than is seen in WT kidneys. Proliferation occurs in NP cells, which are maintained longer than in WT kidneys, and they keep producing new nephrons. In the future, it will be interesting to see whether it will be possible to administer excess GDNF in a manner favorable for continued postnatal nephrogenesis without severely affecting UB morphogenesis to distort the overall organ growth.

Our data demonstrate that the early decline in embryonic NPs of *Gdnf*^hyper/hyper^ kidneys is due to decreased cell proliferation rather than increased premature differentiation. This cellular mechanism is supported by molecular changes detected in known GDNF targets (*Crlf1*, *Wnt11* and *Srgn*) (Lu et al., 2009; Ola et al., 2011) and in important regulators of NP cell maintenance signaling (*Wnt9b*, *Wnt11* and their transcriptional targets) (Karner et al., 2011; Kiefer et al., 2012; O'Brien et al., 2018). Previous examination of longevity and resilience of NP cells by ablating the NP population with diphtheria toxin A, or by disturbing MAPK/ERK activation, which both resulted in rapid decline in NP cells without a positive effect on their lifespan (Cebrián et al., 2014; Ihermann-Hella et al., 2018), together with our results suggest that NP cell proliferation inherently plays a major role in defining their total lifetime. In agreement, NP cells in late embryonic kidneys show significantly reduced proliferation rates and accelerated differentiation, whereas the transplantation of old NP cells into earlier-stage kidneys prolongs their lifespan (Chen et al., 2015a).

**Fig. 9.**
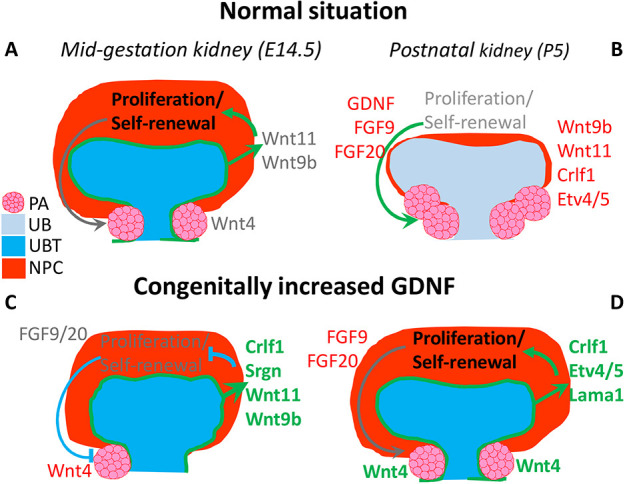
**Schematic of the effects of excess GDNF on developing mouse kidney.** (A) In embryonic kidney undergoing active growth, the nephron progenitor cells (NPC, red) receive secreted guidance signals (e.g. *Wnt11* and *Wnt9b*) from ureteric bud tips (UBT, dark blue) to control the balance of NP proliferation and self-renewal and their differentiation into pretubular aggregates (PA, pink cell cluster). (B) In postnatal kidneys, the tip identity of the UB (UB, light blue) is lost, as molecularly evidenced by the decline in the expression of e.g. *Wnt9b*, *Wnt11*, *Crlf1* and *Etv4*/*5*, indicated by thin red letters. Gradual loss of the UB-derived secreted factors (*Wnt9b*, *Wnt11* and *Crlf1*) contributes to the wane of the NP proliferation and self-renewal. Concomitantly, GDNF and FGF levels decrease, which coincides with accelerated NP differentiation. These together deplete the NP pool quickly resulting in cessation of nephrogenesis. (C) Excess GDNF in embryonic kidneys induces abnormal UBT morphology and augments expression of the bud-derived, secreted regulatory molecules (*Crlf1*, *Srgn*, *Wnt11* and *Wnt9b*). Excess expression of bud-derived secreted molecules has an inhibitory effect on the NP cell cycle resulting in reduced proliferation and self-renewal. Drop in overall NP numbers slows down PA differentiation, as detected by diminished *Wnt4* expression. (D) In the postnatal kidneys, the UBT morphology, and thus the niche it provides for the NPs, normalizes despite the higher than WT expression of bud-derived secreted targets (*Crlf1*, *Etv4*/*5* and *Lama1*). Together with normalized *Wnt9b* and *Wnt11* (not shown), these molecular changes subsidize normal NP proliferation, self-renewal and differentiation, as evidenced by the maintenance of a large NP population, higher *Wnt4* expression and similar amount of nephron precursors (pink clusters of cells) as in WT embryonic kidneys. Through such mechanisms, constitutive GDNF excess under its own promoter enables the nephrogenic program to continue until P7. Dark gray text, non-changed expression; red text, decreased expression; green text, increased expression.

Based on our results, we propose that GDNF contributes to the mechanism through which NP cells sense their developmental age and ultimately their lifespan. In a normal kidney, GDNF expression is high at the initiation of kidney morphogenesis and during the active growth phase, whereas a clear decline in GDNF levels occurs in late embryonic kidneys, resulting in loss of expression by early postnatal stage (Figs 7 and 9). GDNF levels in early *Gdnf*^hyper/hyper^ kidneys are abnormally high, which appears to be inhibitory for NP cell proliferation, likely due to the detected cellular and molecular changes within the mutant UB such as an altered WNT pathway (Fig. 7E,F; Fig. S7A,B), which has known effects on NPC biology (Karner et al., 2011; Kiefer et al., 2012; Nagy et al., 2016; O'Brien et al., 2018). Similar to WT kidneys, *Gdnf* mRNA and GDNF protein levels decrease in postnatal *Gdnf*^hyper/hyper^ kidneys in which *Gdnf* is manipulated at the post-transcriptional level allowing endogenous temporal regulation of the expression (Kumar et al., 2015). Likely, this diminishes GDNF expression to the level that is permissive for NP cell self-renewal and differentiation, which are detected as a sustained nephrogenic program in postnatal kidneys (Fig. 9). Such a newly recognized growth factor-dependent plasticity in renal differentiation bears hope for its use in regenerative purposes in the future.

The first clues for the possibility of lengthening the nephron differentiation period came from experiments inhibiting BMP/SMAD1/5 signaling, which led to slightly bigger kidneys with more nephrons than in vehicle-treated kidneys (Brown et al., 2015). Decreased dosage of *Tsc1*, encoding an mTOR inhibitor, maintains NP cells for an additional day, without reported effects on embryonic NP cell numbers (Volovelsky et al., 2018). Prolonged nephrogenesis was observed also in mice with overexpression of *Lin28*, *Lin28b* or inactivated *Let-7* (*Let-7a1; Let-7d; Let-7f1*) (Urbach et al., 2014; Yermalovich et al., 2019), which also either show direct tumorigenesis or activation of the *Igf2* or *H19h* loci associated with pediatric kidney cancer Wilms' tumor (Chen et al., 2018; Ogawa et al., 1993). As a note, no changes in pSMAD1/5 localization or levels were detected in *Gdnf*^hyper/hyper^ kidneys, which are hypodysplastic and compatible with life for three weeks.

The present study focuses on the characterization of GDNF function in nephrogenesis, which has been previously examined by diminishing endogenous *Gdnf* dosage and results in reduced nephron endowment (Cullen-McEwen et al., 2001, 2003). The canonical receptor complex for GDNF is formed by the RET tyrosine kinase and its co-receptor GFRa1, which are exclusively co-expressed only in the UB epithelium (Costantini, 2012; Golden et al., 1999; Pachnis et al., 1993). GDNF is a key niche factor for UB tip cells (Chi et al., 2009; Riccio et al., 2016; Shakya et al., 2005). We have previously shown that excess GDNF expands collecting duct progenitor pool at the expense of ureteric trunk elongation, resulting in expanded tip and short trunk phenotype due to defects in progenitor motility and shortened cell cycle length (Li et al., 2019). During the embryonic stages the combined effect of abnormally high GDNF, anomalous UB tip morphology-induced biophysical changes and associated molecular alterations in GDNF targets and the WNT pathway appear to jointly push NP cells towards diminished proliferation. We show here that the expanded UB tip morphology ameliorates over the time but persists at mild forms up to P4 in postnatal *Gdnf*^hyper/hyper^ kidneys (Fig. 5). UB tip cells are molecularly still present in P5 *Gdnf*^hyper/hyper^ kidneys, which express high levels of GDNF-regulated tip markers *Crlf1*, *Etv4*, *Etv5* and *Lama1*, NPC-expressed canonical WNT targets *Cited1* and *Uncx4.1*, as well as WNT agonist *R-spondin1* (Fig. 7E,F; Fig. S7A,B) (Karner et al., 2011; Lu et al., 2009; Vidal et al., 2020). Such molecular changes in the postnatal UB likely thus provide a favorable environment for sustained NP self-renewal beyond P2/P3.

We identified UB-derived secreted CRLF1 and WNT11 as GDNF targets that, together with augmented canonical WNT signaling, additionally boosted by increased *Wnt9b* expression, mediate its effects on NP regulation. Our data also indicate that the function of CRLF1 is redundant with other cytokines as its inactivation does not affect renal differentiation. Both WNT9b and WNT11 regulate many aspects of NPCs and nephrogenesis (Carroll et al., 2005; Karner et al., 2011; Majumdar et al., 2003; Nagy et al., 2016; O'Brien et al., 2018). Upregulation of UB-expressed *Wnt9b*, which is known to regulate many aspects of nephrogenesis, in *Gdnf*^hyper/hyper^ kidneys may mimic its forced expression in NPs, which disrupts their maintenance versus differentiation equilibrium (Kiefer et al., 2012), and thus mechanistically contributes to imbalanced NP self-renewal in *Gdnf*^hyper/hyper^ nephrogenesis. This is supported by improved tip morphology upon canonical WNT inhibition by IWR1 in whole kidneys facing excess GDNF.

Our results show that IWR1 inhibition itself has a major effect on NPCs, which are dispersed and loosely adhered to UB tips. Canonical WNT inhibition in NPCs themselves likely contributes to the failure to augment their phenotype in the presence of excess GDNF. This is supported by our genetic experiments in which lowering UB-expressed *Wnt11* expression in the *Gdnf*^hyper/hyper^ background improved NP maintenance and differentiation, but failed to fully rescue the renal hypoplasia caused by the excess GDNF. This may at least partially be because of the failure to inactivate both *Wnt11* alleles in the *Gdnf*^hyper/hyper^ background due to the embryonic lethality of *Wnt11^−/−^* (Nagy et al., 2016). Thus, one remaining *Wnt11* allele may even augment the GDNF cascade as suggested earlier (Majumdar et al., 2003) and thus in part prohibit full rescue of nephrogenesis.

Our current study cannot rule out the possibility that some effects of GDNF on NPs are due to non-canonical GDNF signaling, as its potential alternative signaling receptors are expressed by the NPs (Brodbeck et al., 2004; Enomoto et al., 2004; Keefe Davis et al., 2013; Paratcha et al., 2003). However, both deletion of NCAM or expression of *Gfra1* in UB only (by placing it under the control of the *Ret* promoter) maintain normal renal phenotype (Cremer et al., 1994; Heuckeroth et al., 1999; Keefe Davis et al., 2013; Rossi et al., 1999; Sariola and Saarma, 2003). This suggests that, at least on their own, neither NCAM nor GFRa1, are essential for the nephrogenic program.

In summary, our experiments suggest that GDNF, which has been, and currently is, in clinical trials for Parkinson's disease (Kirkeby and Barker, 2019), may bear potential to serve as a prospective means to improve nephron endowment in the future. Additional experimentation is needed to identify possible means to excessively activate GDNF signaling without harmful effects on kidney growth.

## MATERIALS AND METHODS

### Animals

Animal care and research protocols were performed in accordance with the Code of Ethical Conduct for Animal Experimentation as well as European Union directives (Directive 2010/EU/63), and were approved by the National Animal Experiment Board of Finland.

Mice were housed in individually ventilated cages with food and water *ad libitum*. Optimal humidification and heating were provided constantly at the certified Laboratory Animal Centre of the University of Helsinki.

The generation and genotyping of the *Gdnf*^hyper^ (Kumar et al., 2015) and *Fgf9;20* (Barak et al., 2012) mouse models has been described previously. *Gdnf*^hyper^;*Wnt11*^−^ pups are from intercrosses of *C57BL6 Wnt11^−^* (Nagy et al., 2016) and *Gdnf*^hyper^ lines, and were genotyped as previously described (Kumar et al., 2015; Nagy et al., 2016). All the mouse lines used in the studies were maintained on a 129Ola/ICR/C57bl6 triple mixed background.

The generation of *Crlf* knockout mice has been described previously (Alexander et al., 1999). PCR genotyping was performed using a Neo-specific primer set (5′-AGAGGCTATTCGGCTATGACTG-3′ and 5′-CCTGATCGACAAGACCGGCTTC-3′) together with gene-specific primer sets for *Crlf1* (5′-GCCTAATAGGTGCTGGGTGA-3′ and 5′-GACCCTATCTGCGTTTTCCA-3′).

### Tissue collection

Embryos were staged according to the criteria of Theiler (1989) as described previously (Kuure et al., 2005) and collected after cervical dislocation of pregnant females at deep anesthesia with CO_2_. Late embryonic and postnatal pups were sacrificed via decapitation. Tissue was dissected in Dulbecco's medium supplemented with 0.2% bovine serum albumin (BSA) followed by fixation with 4% paraformaldehyde (PFA) or organ culture.

### Organ culture

Kidneys isolated from E11.5 and E12.5 mouse embryos were placed at the air-liquid interface supported by Transwell^®^ polyester membrane system (Costar) and cultured at 37°C in a humidified 5% CO_2_ atmosphere with kidney culture medium [F12:DMEM (1:1)+Glutamax (Gibco)] supplemented with 10% fetal bovine serum and penicillin-streptomycin (Ihermann-Hella and Kuure, 2019). For the *in vitro* culture with 100 ng/ml exogenous GDNF (ProSpec Ltd) (Sainio et al., 1997) aimed at NPC quantification, each urogenital block containing metanephros, the definitive kidney rudiment, was halved along the linea mediana ventralis. One-half of each sample was cultured with exogenous GDNF while the other half served as the control. WNT inhibition experiments were first tested with 50-100 ng/ml GDNF and 50-100 µM IWR1 (I0131-25, Sigma-Aldrich). Based on these dose dependence experiments the optimal concentrations were 50 ng/ml GDNF and 75 µM IWR1.

### Histology and immunostaining

For studies investigating the marker of interest on paraffin sections, kidneys were dissected from the embryos or pups of the indicated stages, followed by tissue processing and sectioning as previously described (Li et al., 2019). A minimum of five sections from each kidney and three to five different kidneys per genotype were used in each analysis. The exact sample numbers are provided in the figure legends.

Hematoxylin and Eosin (H&E) and immunohistochemistry performed on paraffin sections were completed as previously described (Li et al., 2019). In brief, the dissected tissue was fixed overnight with 4% PFA (pH 7.4), followed by dehydration and paraffinization with an automatic tissue processor (Leica ASP 200). Sections of 5 µm thickness were prepared and dewaxed in Xylene followed by rehydration before being stained with Harris H&E or undergoing heat-induced antigen retrieval in antigen retrieving buffer (10 mM Citrate; pH 6.0). For immunostaining, 3% BSA was used for blocking (1 h at room temperature) followed by 30 min 0.5% hydrogen peroxide treatment for chromogenic staining. Sections were then incubated with primary antibodies overnight at +4°C followed by species-specific secondary antibody incubation for 2 h at room temperature. Chromogenic detection was implemented with EnVision Detection System-HRP (DAB) kit (Dako).

E11.5 and E12.5 kidneys subjected to whole-mount immunofluorescence staining were fixed and permeabilized with 100% ice-cold methanol for 10 min before overnight incubation with primary antibodies. Incubation with the corresponding species-specific secondary antibodies was implemented on the second day following a vigorous wash with PBST (0.1% Tween 20 in PBS). Details about the primary and secondary antibodies used in the present study are listed in Table S1.

### *In situ* hybridization

*In situ* hybridization was conducted as previously described (Ihermann-Hella et al., 2014). In brief, an anti-sense RNA probe against *Gdnf* exons was transcribed and hybridized on thick sections derived from P4 kidneys (10-15 sections/kidney, *n*=3 kidneys/genotype) embedded in 4% low melting agarose (NuSieve GTG, Lonza). BM Purple was used for colorimetric reaction.

### ELISA

GDNF protein levels in P7.5 kidneys were measured via ELISA as reported previously (Kumar et al., 2015). GDNF Emax^®^ Immunoassay (Promega) was used with acid treatment when carrying out the ELISA.

In detail, for each genotype, three kidneys were lysed immediately after being dissected. After measuring the protein concentration with DC protein Assay (Bio-Rad), 25 µg of total protein was loaded into the well on ELISA plate. Each sample was analyzed in duplicate.

### Imaging

Immunostaining and *in situ* hybridization on sections were imaged using either a Zeiss AxioImager equipped with the HXP 120V fluorescence light source, a Hamamatsu Orca Flash 4.0 LT B&W camera and Zeiss AxioCam 105 color camera, or with a Leica DM5000B equipped with Leica EL 6000 metal halide light source and Hamamatsu Orca-Flash4.0 V2 sCMOS camera. Whole-mount immunofluorescence staining was imaged using a Zeiss AxioImager with the Zeiss ApoTome application. Whole-mount imaging for intact kidney size measurements was performed using a Leica MZFLIII equipped with a Leica DFC7000T camera.

### Quantitative analyses based on immunostaining and imaging

Quantification of the glomerular density was calculated from the sequential sections of E18.5 and P7 kidneys. The kidneys were sectioned through, and sections for glomerular quantification were picked every 125 µm to avoid iteration count of glomeruli. The number of glomeruli in each section were counted manually and the area of each kidney was measured with Fiji ImageJ software (V1.51) through outlining the contour manually. To calculate the glomerular densities, the total number of glomeruli and number of glomeruli in the cortex were counted separately. The average total kidney area and overall and cortical glomeruli numbers were then calculated for each sample. The average glomerular density was calculated by dividing the average glomerular number by the average measured area. The average of cortical glomeruli within the entire average area was calculated by dividing the average cortical glomerular number by the average measured total area. Only glomeruli with intact shape were recorded.

Quantification of the total SIX2-positive NPs at E11.5 (*n*=4 for WT, *n*=5 for *Gdnf*^hyper/hyper^) and E12.5 (*n*=10 kidneys/treatment, three independent experiments) were based on the whole-mount immunofluorescence staining. The images were captured with the application of Zeiss ApoTome and then processed with Imaris Software (Bitplane) using spot tracking. All images were processed with identical analysis protocols. In order to normalize the variation between the litters, the mean quantity of SIX2-positive NPs in the WT kidneys within each litter was set to 100%, and the quantity of SIX2-positive NPs in the *Gdnf*^hyper/hyper^ kidneys was compared with the mean quantity in the WT kidneys within the litter.

The mitotic index of SIX2-positive NPs in E12.5 kidneys (*n*=10 kidneys/treatment, three independent experiments) cultured with exogenous GDNF were calculated in a similar way to normalize the variation between litters. The mean proportion of proliferating SIX2-positive NPs in the WT kidneys and kidneys cultured without exogenous GNDF within each litter was set to 100% and the proportion of proliferating SIX2-positive NPs in the presence of excess GDNF (*Gdnf*^hyper/hyper^ kidneys and exogenous application) from the same litter was plotted in the figure after comparing with the WT littermates. The mitotic index of SIX2-positive NPs in E11.5 kidneys cultured with exogenous GDNF were plotted as the original data/value.

The calculation of the average SIX2-positive NPs per nephrogenic niche (E14.5 and P0) and nephrogenic niches with progenitors (P3-P6) was conducted by immunofluorescence staining in paraffin sections. The number of SIX2-positive NPs was counted with Fiji ImageJ software (V1.51) on each section selected for the analysis using particle analysis and the number of tips was counted manually on the corresponding sections. The number of analyzed samples at E14.5 was three kidneys for WT and *Gdnf*^hyper/hyper^ kidneys and two kidneys for *Gdnf*^hyper/hyper^*;Wnt11^+/−^*. The number of tips analyzed at E14.5 was 207 in WT, 431 in *Gdnf*^hyper/hyper^ and 107 for *Gdnf*^hyper/hyper^*;Wnt11^+/−^* kidneys. The number of analyzed samples at P0 was three kidneys per genotype, and number of tips analyzed, which is presented in the corresponding figures, varies from 10 to 315, being lowest in WT P6 kidneys, in which the tips are rarely present.

UB tip measurements were carried out using the LAS X program of the Leica microscope operating system after 48 h culture in the presence of specified chemicals and visualization of the tips with E-cadherin antibody (Table S1). The tips were measured from six kidneys in each condition of two independent set of experiments in which the number of tips measured was: 101 for GDNF, 131 for IWR1 and 115 for 75 µM IWR1+50 ng/ml GDNF.

The kidney size measurements in experiments assessing the effect of *Wnt11* deletion in *Gdnf*^hyper/hyper^ background were conducted with Fiji ImageJ software (V1.51) on whole-mount images through manual tracing of the contour of the kidney.

### Statistical analysis

All values are presented as mean±s.e.m. except the main effects analysis of *Gdnf* and *Wnt11* on kidney size, which are shown as mean± s.d. Statistical significance was set at *P*<0.05 for all the analysis. Independent-samples two-tailed *t*-test was used for pairwise comparisons with SPSS Statistics software (IBM; Version 25) in the study unless otherwise noted. The data obtained via *in vitro* tissue culture with exogenous GDNF was analyzed with the paired-sample *t*-test using SPSS Statistics software. The impact of genetically modified *Gdnf* and *Wnt11* expression on kidney size was analyzed via two-way ANOVA followed by main effects test with SPSS Statistics software. The statistical significance for the deviation of gene distribution from Mendelian ratio was performed using χ^2^ test also with SPSS Statistics software. The data obtained from ELISA, qRT-PCR and WNT inhibition experiments were analyzed with one-way ANOVA followed by Bonferroni *post-hoc* test using SPSS Statistics software.

### Reverse transcription and quantitative RT-PCR

Quantitative RT-PCR (qRT-PCR) experiments were conducted as previously reported (Kumar et al., 2015). In more detail, 150-500 ng of total RNA from each sample (*n*=3 kidneys/genotype at given stage) was treated with RNase-free DNase I (Thermo Fisher Scientific). The reverse transcription reaction was performed with random hexamer primers and RevertAid Reverse Transcriptase (Thermo Fisher Scientific) immediately after inactivation of DNase I with 5 mM EDTA at 65°C. Complementary DNA (cDNA) was diluted 10× and stored at −20°C until qRT-PCR.

For qRT-PCR, LightCycler 480 SYBR Green I Master (Roche) or Bio-Rad C1000 Touch Thermal Cycler upgraded to CFX384 System (Bio-Rad), supplied with SYBR Green I Master (Roche) and 250 pmol primers was loaded into the well of 384-well plates with a total volume of 10 µl. Negative control was always included in every reaction via setting conditions with minus-reverse transcription or water. A combination of *Pgk1*, *Hprt* and *Gapdh* was used as the reference genes in all the qRT-PCR experiments, except the one with P7.5 kidneys, in which mouse *Actb* was reference gene. Primer sequences can be found in Table S2.

Data obtained from qRT-PCR were analyzed as described previously (Kumar et al., 2015). Normalization was performed according to the geometric mean of the reference genes. All samples were analyzed in a duplicate manner. Results for a biological repeat were discarded when the Cq value for one or more of the replicates was 40 or 0, or when the Cq difference between replicates was >1.

## Supplementary Material

Supplementary information

Reviewer comments
