# Supplementary material for: Postnatal prolongation of mammalian nephrogenesis by excess fetal GDNF
Source: Development. 2021 May 25;148(10):dev197475. doi: 10.1242/dev.197475 (PMC8180252; doi:10.1242/dev.197475)
Supplement: Supplementary information [file develop-148-197475-s1.pdf]

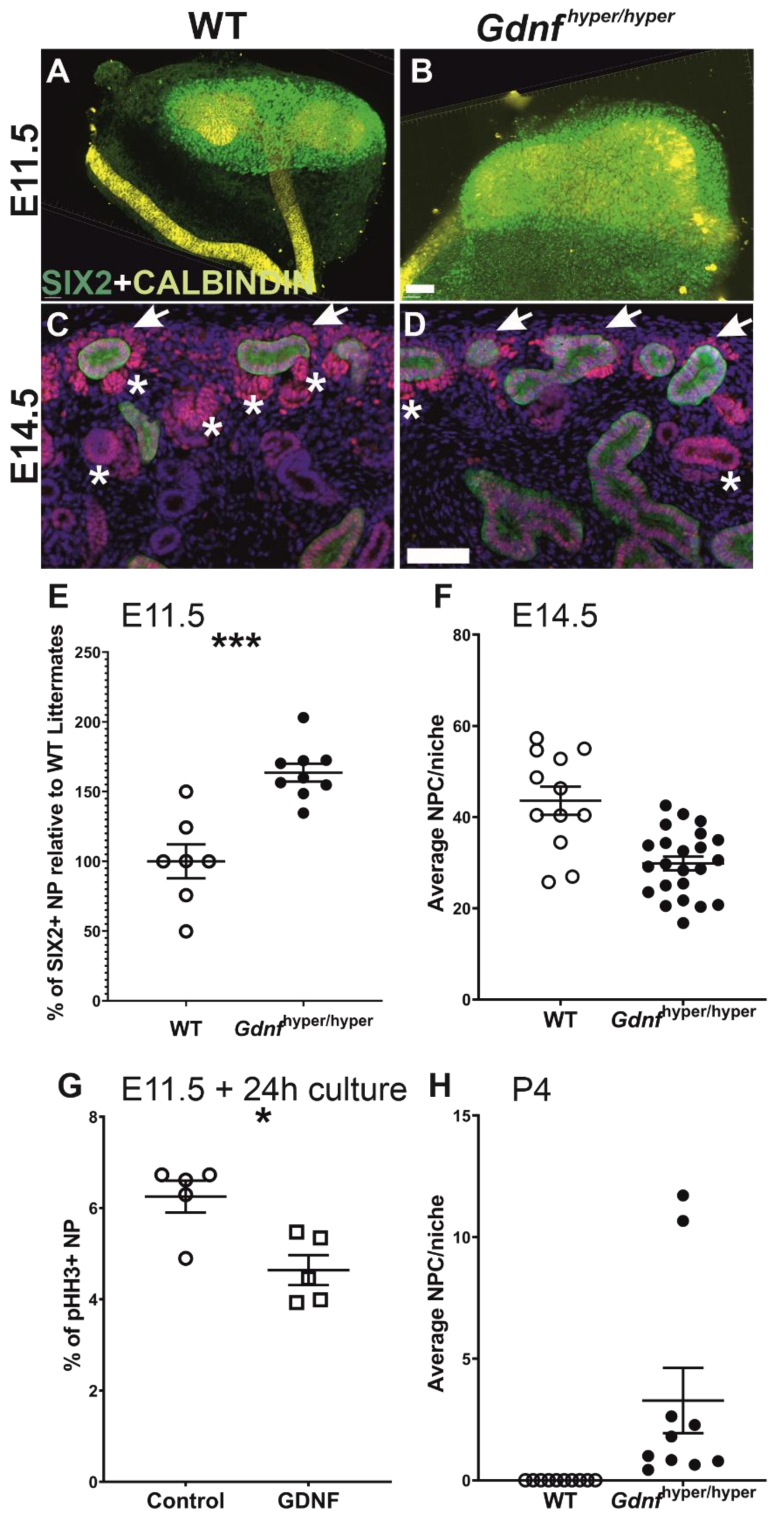

Figure S1. Decreased nephron progenitor cell numbers and proliferation in *Gdnf*$^{hyper/hyper}$ kidneys. (A) Three-dimensional reconstructed apotome z-stacked images of representative E11.5 WT and (B)

Development • Supplementary information

Gdnf$^{hyper/hyper}$ kidneys stained with CALBINDIN (yellow) and SIX2 (green). (C) PAX2 (red) and E-CADHERIN (green) localization in wild type (WT) and (B) *Gdnf*$^{hyper/hyper}$ kidneys at E14.5 shows decrease in nephron progenitors (NP, arrows) and enlargement of ureteric bud epithelium (green). Asterisks mark the differentiating nephron precursors, which are greatly reduced in *Gdnf*$^{hyper/hyper}$ kidneys. For each staining at given stage n=3 kidneys/genotype were used. (E) Comparison of SIX2-positive NP amount in WT and *Gdnf*$^{hyper/hyper}$ kidneys shows increased initial NP pool in *Gdnf*$^{hyper/hyper}$ kidneys at E11.5. Data are presented as mean quantity of SIX2-positive NPs ± SEM compared with those of WT littermates, which are set to 100% and reflect the results obtained from four independent litters (WT: 100 ± 12.15 %, n=7; *Gdnf*$^{hyper/hyper}$: 163.59 ± 6.42 %, n=9; P=0.000). (F) Scatter plots showing the quantification of NPC numbers at E14.5. Data is collected for 207 WT and 431 *Gdnf*$^{hyper/hyper}$ UBs, which had total of 8805 and 12696 NPCs, respectively (n=4 kidneys for WT; n=8 kidneys for *Gdnf*$^{hyper/hyper}$). (G) Proportion of proliferating SIX2-positive NPs in E11.5 kidneys cultured for 24 hours with or without (control) 100 ng/mL GDNF supplementation. (Control: 6.25 ± 0.34 %, n=5; GDNF: 4.64 ± 0.33 %, n=5; P=0.012). (H) Scatter plots showing the quantification of NPC numbers at P4. Data is collected for 315 WT and 245 *Gdnf*$^{hyper/hyper}$ UBs, which had total of 0 and 462 NPCs, respectively (n=4 kidneys for WT; n= 2 kidneys for *Gdnf*$^{hyper/hyper}$). *Scale bar*: 100µm.

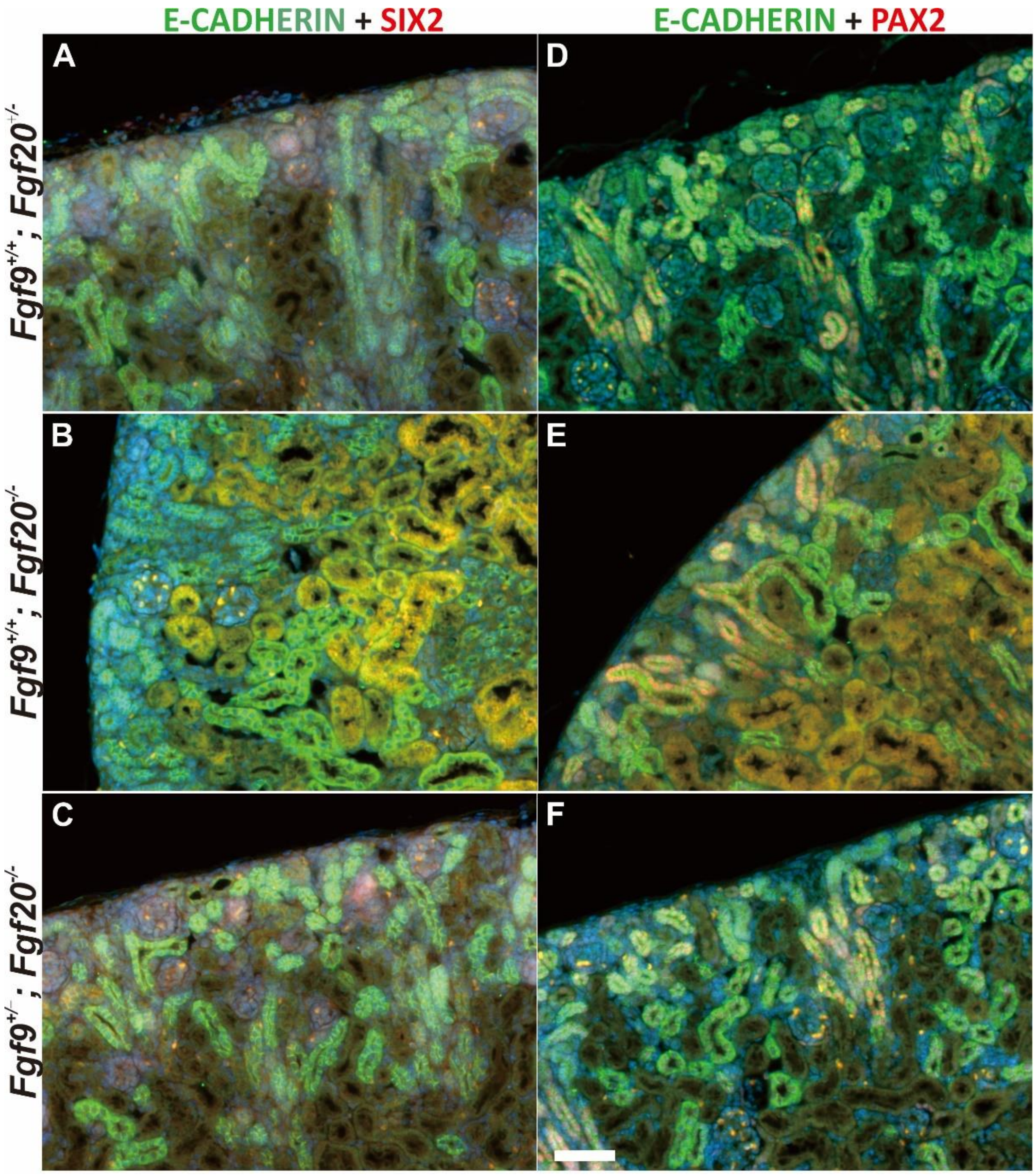

Figure S2. Nephron progenitors are not maintained in postnatal kidneys upon *Fgf9* and *Fgf20* deletion. Representative images showing SIX2 (red) and E-CADHERIN (green) staining in (A) $Fgf20^{+/-}$ (n=3), (B)"$Fgf20^{-/-}$ (n=4) and (C) $Fgf9^{+/-}$;$Fgf20^{/-}$ (n=4) kidneys at P6. (D) PAX2 (red) and E-CADHERIN"(green) staining in $Fgf20^{+/-}$ (n=3), (E) $Fgf20^{-/-}$ (n=4) and (F) $Fgf9^{+/-}$;$Fgf20^{-/-}$ (n=4) kidneys at P6 all indicate similar result. Cap mesenchyme that would be positive for SIX2 or PAX2, indicating the presence of nephron progenitors and differentiating precursors, cannot be detected in any of the FGF-deficient kidneys. *Scale bar*: 50µm.

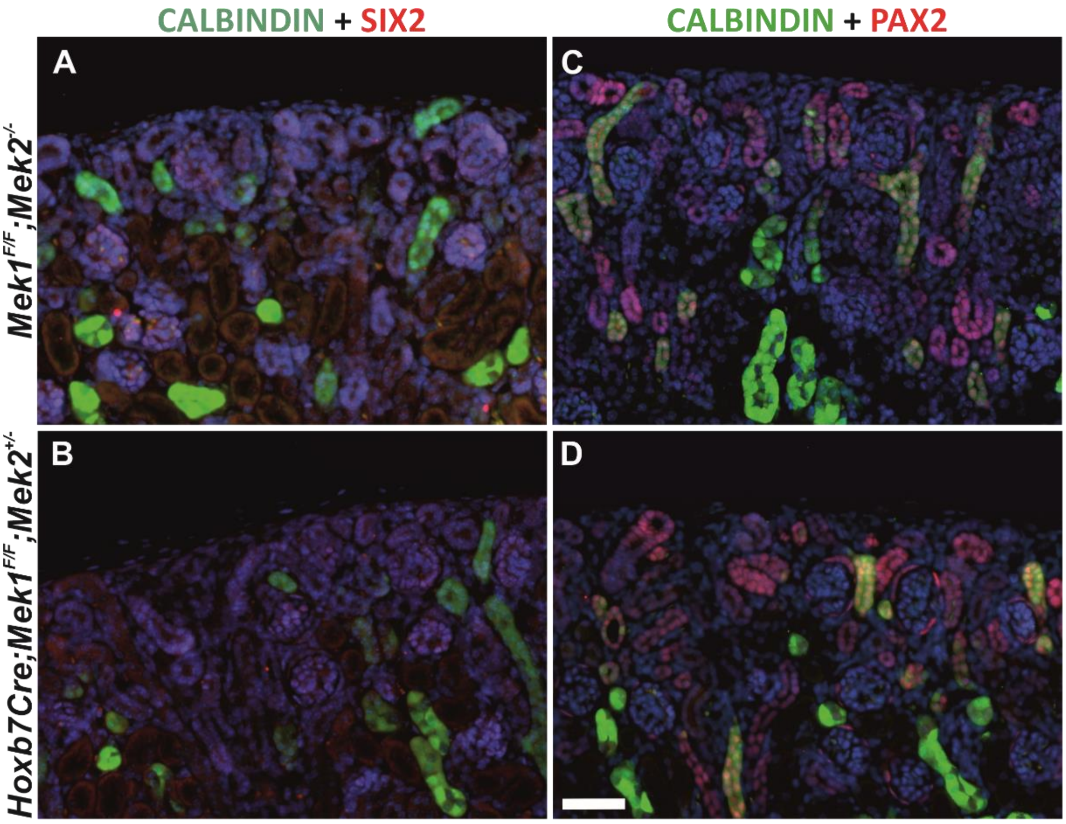

Figure S3. Nephron progenitors are not maintained in Mek1/2-deficient postnatal kidneys. Representative images showing SIX2 (red) and E-CADHERIN (green) staining in (A) *Mek2*$^{-/-}$ (n=2) and (B) *Hoxb7Cre;Mek1*$^{fl/fl}$*;Mek2*$^{+/-}$ (n=2) kidneys at P5. (C) PAX2 (red) and E-CADHERIN (green) staining in *Mek2*$^{-/-}$ (n=2) and (D) *Hoxb7Cre;Mek1*$^{fl/fl}$*;Mek2*$^{+/-}$ (n=2) kidneys at P5. Cap mesenchyme that would be positive for SIX2 or PAX2, indicating the presence of nephron progenitors and differentiating precursors, cannot be detected in any of the MEK-deficient kidneys. *Scale bar*: 50µm.

Development • Supplementary information

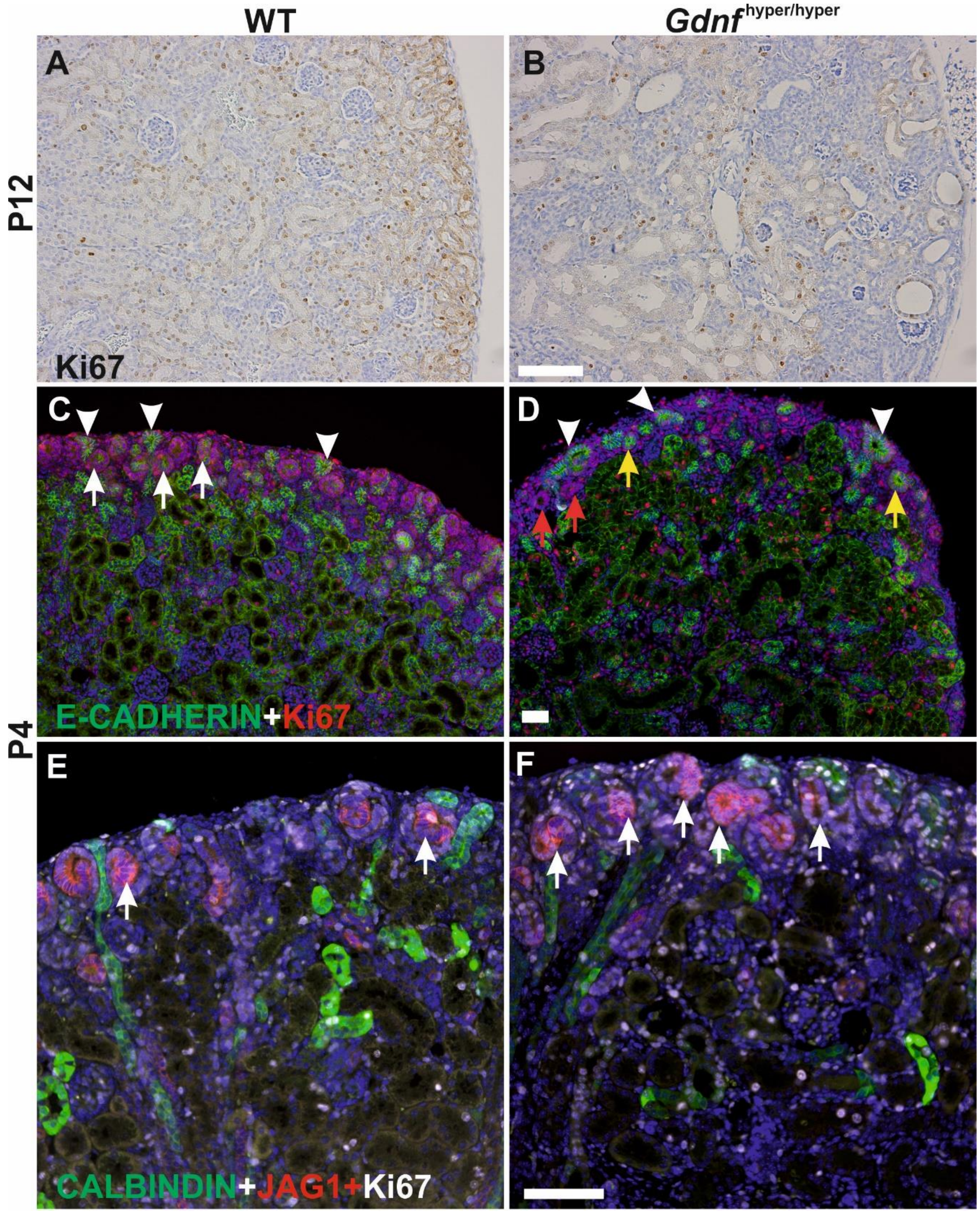

Figure S4. Sustained cortical proliferation in postnatal nephrogenesis in *Gdnf*$^{hyper/hyper}$ kidneys. (A) Ki67 staining visualizes similar pattern of proliferative cells in P12 WT and (B) *Gdnf*$^{hyper/hyper}$ kidneys (n=2 kidneys/genotype). (C) Co-staining of E-CADHERIN (green) and Ki67 (red) in P4 WT and (D) *Gdnf*$^{hyper/hyper}$ kidneys (n=3 kidneys/genotype). White arrows point to nephron precursors, which are double positive for Ki67 and E-CADHERIN, red arrows indicate newly induced renal vesicles, which have not yet have acquired the expression of E-CADHERIN.

Development • Supplementary information

Yellow arrows point to distal tubules of nephrons positive for Ki67 and abnormally positioned to renal cortex. Arrowheads indicate ureteric buds, which also show proliferative activity. (E) P4 WT kidneys (n=4) stained with JAG1 (red), CALBINDIN (green) and Ki67 (white) shows proliferation in differentiating nephron precursors (arrows). (F) Corresponding triple staining in *Gdnf*$^{hyper/hyper}$ kidneys (n=4) demonstrates slightly more on-going nephrogenesis (arrows) but similar proliferation pattern. *Scale bar*: 100µm.

Development • Supplementary information

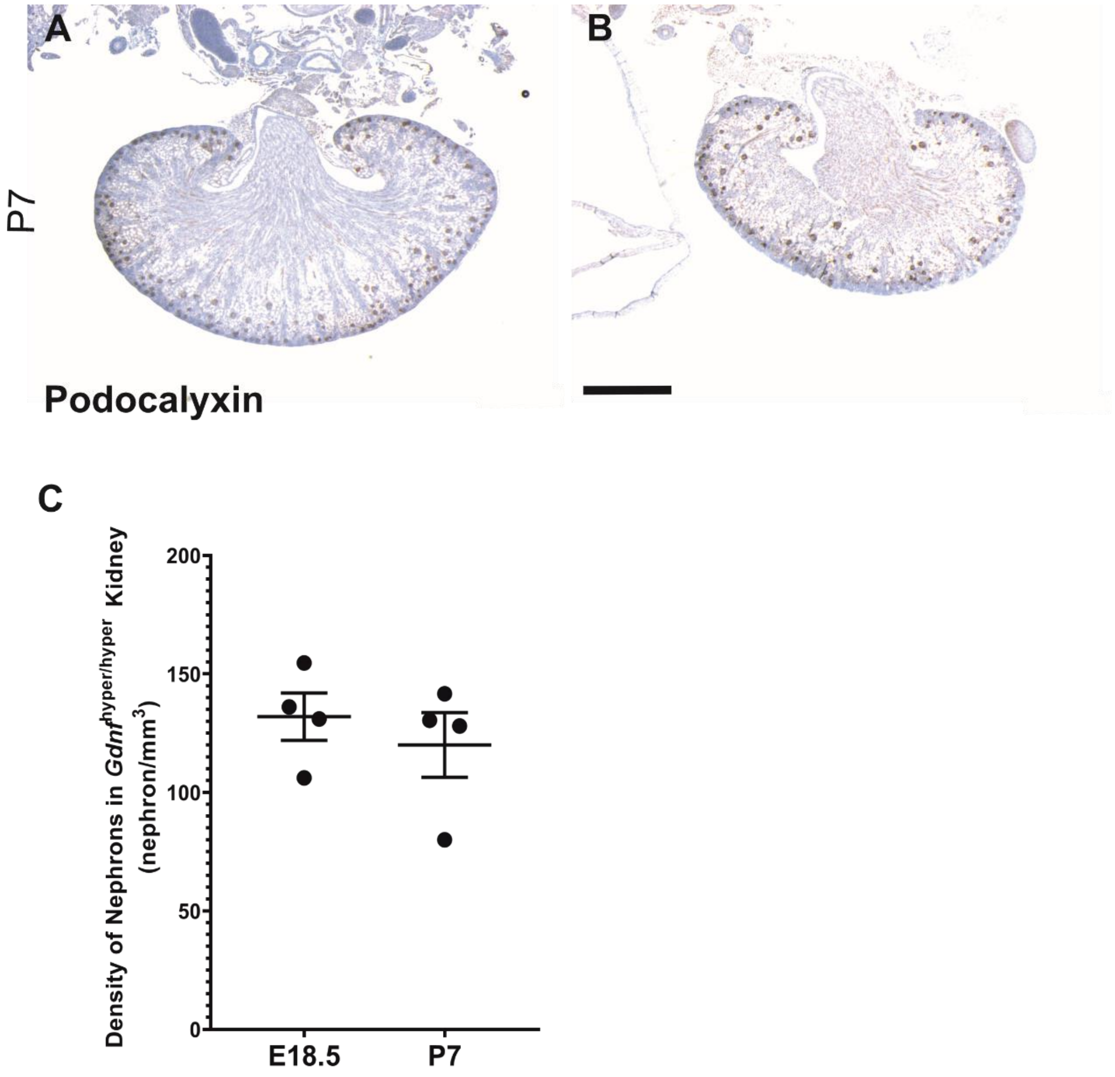

Figure S5. Glomerular density assessment. Representative images showing the distribution of glomeruli in P7 WT kidney (A) and *Gdnf*$^{hyper/hyper}$ kidneys (B) (n=6 kidneys for WT; n=4 kidneys for *Gdnf*$^{hyper/hyper}$). Chromogenic antibody staining was performed for PODOCALYXIN and counterstained with hematoxylin. (C) Scatter plots of nephron density in *Gdnf*$^{hyper/hyper}$ kidneys at E18.5 and P7. Data are presented as the mean quantity of nephron per cubic millimeter ± SEM. (n=4 kidneys/genotype). *Scale bar*: 1mm.

Development • Supplementary information

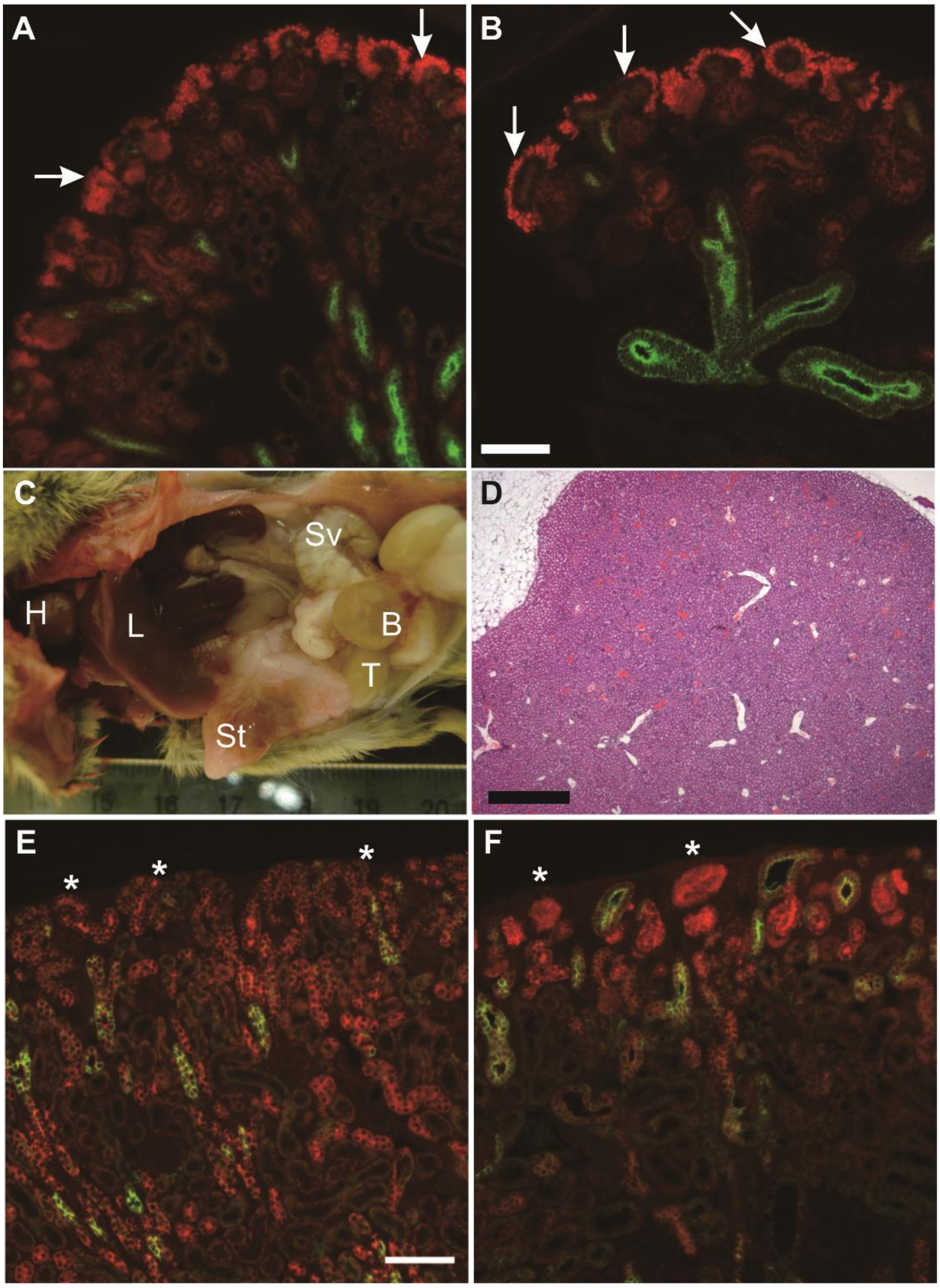

Figure S6. *Gdnf*$^{wt/hyper}$ animals show mildly diminished embryonic nephron progenitor count without consequences on postnatal nephrogenesis. (A) Wild type and (B) *Gdnf*$^{wt/hyper}$ kidneys at E16.5 stained with SIX2 (red), to detect nephron progenitors, and keratin 8 (green) to visualize collecting duct. Arrows point to nephron progenitors, which appear as a thinner layer in Gdnfwt/hyper kidneys indicating their diminished amount. (C) Abdominal cavity of an adult Gdnfwt/hyper mouse at the age of 7 months shows no signs of tumors or other abnormalities. B: bladder, H: heart, L: liver, St: stomach, Sv: seminal vesicle. (D) Histology of

Development • Supplementary information

Gdnf$^{wt/hyper}$ kidney at the age of 4 months shows no neoplasia or tumorigenic growth. (E) Wild type and (F) *Gdnf*$^{wt/hyper}$ kidneys at P5 stained with SIX2 (red), to detect nephron progenitors, and keratin 8 (green) to visualize collecting duct. Asterisks indicate loss of SIX2-positive nephron progenitors in both genotypes.

Development • Supplementary information

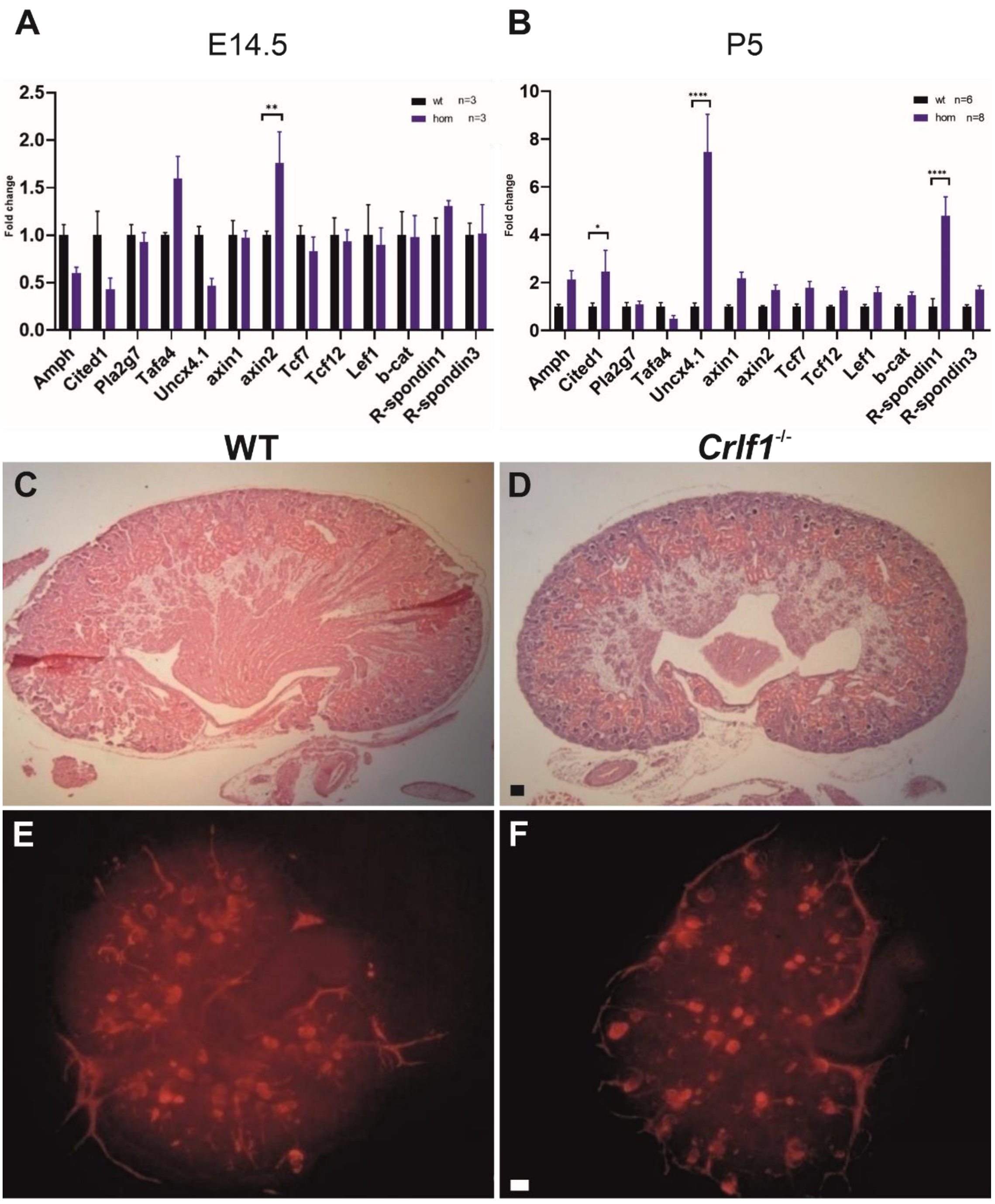

Development • Supplementary information

Figure S7. Gene regulatory networks mediating GDNF functions to nephron progenitors. (A) qRT-PCR based quantification of the relative expression levels of canonical WNT signaling targets at E14 (n=3 kidneys/genotype) and (B) at P5 (n=6 for WT; n=8 for *Gdnf*$^{hyper/hyper}$). Embryonic *Gdnf*$^{hyper/hyper}$ kidneys (E14.5) show significant upregulation of canonical WNT signaling target *Axin2*, while the nephron progenitor specific targets *Amph*, *Cited1* and *Unx4.1* are slightly downregulated. Postnatal *Gdnf*$^{hyper/hyper}$ kidneys (P5) have increased expression of selected nephron progenitor specific WNT targets (*Cited1* & *Unc4.1*) and significantly more expression of *R-spondin1*. (C) Analysis of kidney morphology with hematoxylin and eosin staining at P0 WT and (D) *Crlf1*$^{-/-}$ kidneys (n=15) revealed normal kidney size and organization. (E) PODOCALYXIN immunofluorescence visualizes developing glomeruli in E11.5 WT and (F) *Crlf*1$^{-/-}$ kidneys (n=6/genotype) cultured for 4 days. The average number of nephrons was 34±10 in *Clf1* -/- vs. 29±8 in WT kidneys (p=0.34). *Scale bar*: 100 µm.

Development • Supplementary information

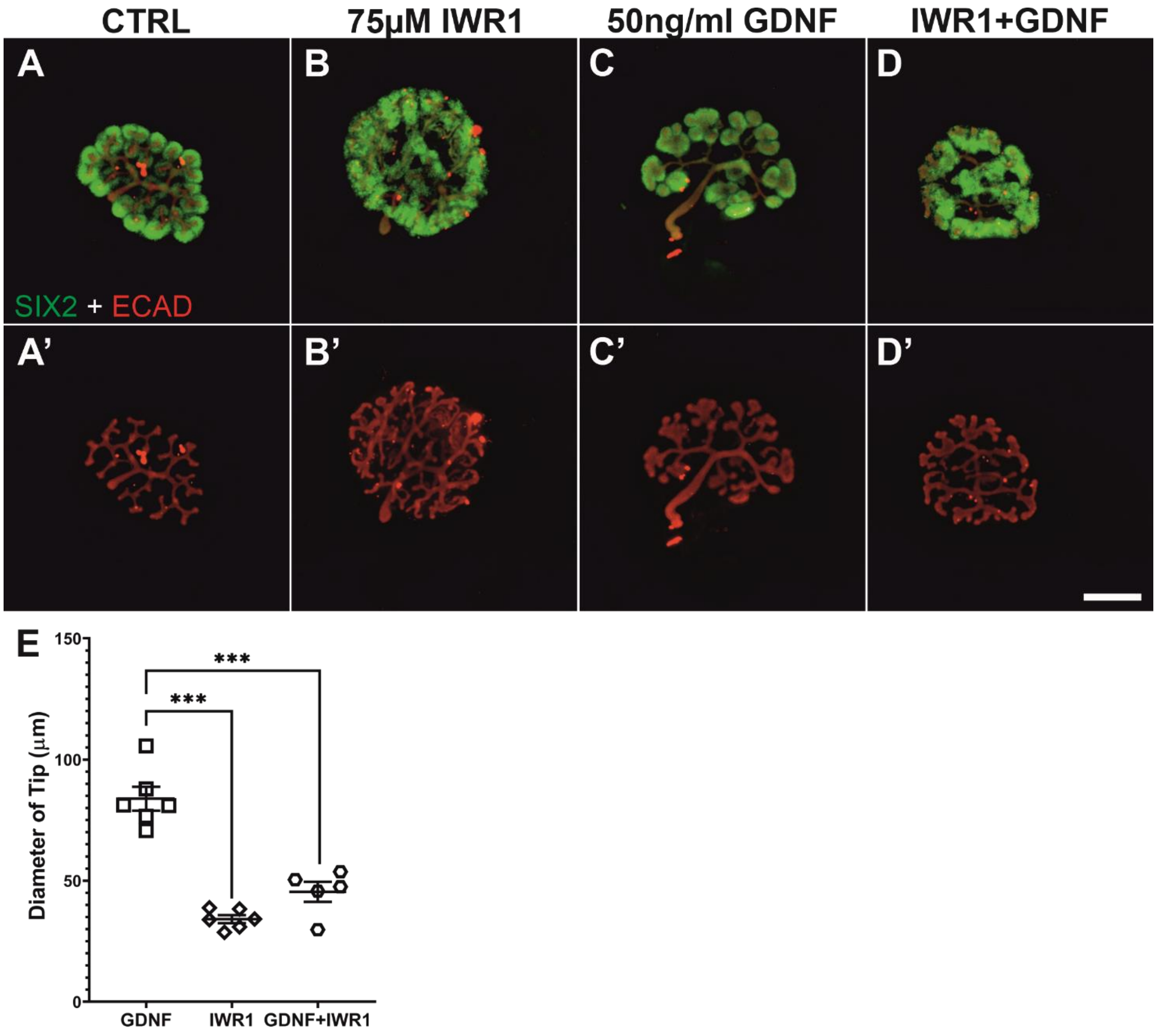

Figure S8. WNT inhibition alleviates GDNF's effect on ureteric bud tip morphology. E12.5 kidneys cultured for 48h in (A) DMSO (Ctrl), (B) 75µM tankyrase inhibitor (IWR1), which prevents canonical WNT signaling, (C) 50ng/ml GDNF and (D) 75µM IWR1+ 50ng/ml GDNF. SIX2 (green) visualizes nephron progenitors and E-cadherin (ECAD, red) labels ureteric bud epithelium. Control kidney (DMSO) shows typical ureteric bud branching morphogenesis and intimate localization of nephron progenitors around each bud tips. WNT inhibition (IWR1) has minor effect on ureteric bud tip morphology but instead disperses nephron progenitors to spatially wider and loser patterns. GDNF treatment mimics genetic increase seen in *Gdnf*$^{hyper/hyper}$

Development • Supplementary information

kidneys where ureteric bud tips are severely expanded. Simultaneous exogenous GDNF and WNT inhibition rescues ureteric bud tip morphology while nephron progenitors are still incorrectly localized but appear more adherent to each other than in IWR1 only treated kidneys. (E) Quantification of tip diameter in IWR1, GDNF and IWR1+GDNF treated kidneys exemplified in A-D (n= 6 / treatment; tips measured: GDNF: 101, IWR1: 131, 75µM IWR1+ 50ng/ml GDNF: 115) shows significant normalization of ureteric bud tip morphology upon IWR1 treatment in kidneys cultured with excess GDNF. *Scale bar*: 400µm.

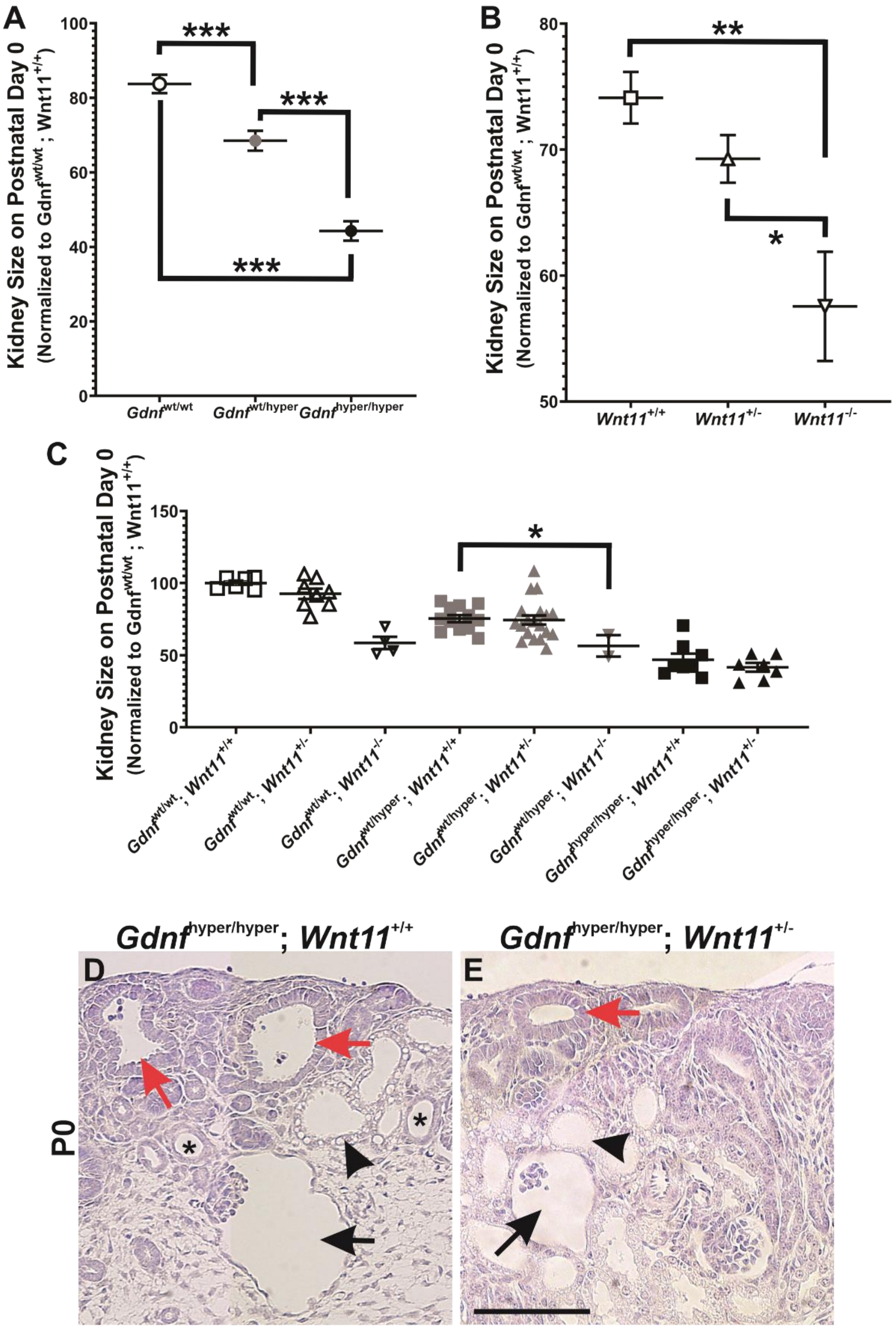

Figure S9. The effect of *Wnt11* deletion in GDNF-hypermorphic kidneys. (A) Main effect analysis of genetically increased GDNF on kidney size in *Gdnf*^{hyper};*Wnt11* compound background. Data are presented as estimated marginal means of area obtained via measuring the

Development • Supplementary information

coronal plane of each kidney ± SD compared to those of *Gdnf*$^{wt/wt}$;*Wnt11*$^{+/+}$ controls and reflect the results obtained from five independent litters. The (mean) size of *Gdnf*$^{wt/wt}$;*Wnt11*$^{+/+}$ kidney(s) in each litter was set to 100% in order to normalize the difference among litters. (B) Main effect analysis of genetically decreased *WNT11* on kidney size in Gdnfhyper;*Wnt11* compound background. Similar analysis was performed as in A. (C) Presentation of size analysis as whole. Data are presented as means of area obtained by measuring the coronal plane of each kidney ±SEM in comparison to *Gdnf*$^{wt/wt}$;*Wnt11*$^{+/+}$ controls, which are set to 100% and reflect the results obtained from five independent litters. (*Gdnf*$^{wt/wt}$;*Wnt11*$^{+/+}$: 100 ± 4.09%, n=6 kidneys; *Gdnf*$^{wt/wt}$;Wnt11+/-: 92.61 ± 3.53%, n=8 kidneys; Gd*nf*$^{wt/wt}$;*Wnt11*$^{-/-}$: 58.58 ± 4.25%, n=4 kidneys; *Gdnf*$^{wt/hyper}$;*Wnt11*$^{+/+}$: 75.45 ± 2.44%, n=12 kidneys; *Gdnf*$^{wt/hyper}$;*Wnt11*$^{+/-}$: 73.52 ± 2.74%, n=19 kidneys; *Gdnf*$^{wt/hyper}$;*Wnt11*$^{-/-}$: 56.52 ± 7.47%, n=2 kidneys; *Gdnfhyper/hyper*;*Wnt11*$^{+/+}$: 46.90 ± 4.16%, n=8 kidneys; *Gdnf*$^{hyper/hyper}$;Wnt11 +/-: 41.67 ± 3.06%, n=7 kidneys). The estimated marginal means of kidney size in (A) and (B) are results of the analysis containing all the data about kidney size in (C). Kidney size in *Gdnf*$^{wt/hyper}$; *Wnt11*$^{-/-}$ is significantly smaller than that in *Gdnf*$^{wt/hyper}$; *Wnt11*$^{wt/wt}$ mutants (P=0.014, unpaired 2-tailed t-test). (D) Representative H&E staining image showing morphology in cortical region of *Gdnf*$^{hyper/hyper}$;*Wnt11*$^{+/+}$ and (E) *Gdnf*$^{hyper/hyper}$; *Wnt11*$^{+/-}$ kidney at P0 (n=3 kidneys/genotype). The dilated UB (red arrow), collecting duct cysts (black arrow head), and cysts in Bowman's space (black arrow) are slightly improved in *Gdnf*$^{hyper/hyper}$;*Wnt11*$^{+/-}$ kidneys than those seen in *Gdnf*$^{hyper/hyper}$. * denotes $P < 0.05$, ** denotes $P < 0.01$, *** denotes $P < 0.001$ in comparison with each other; one-way ANOVA followed by Bonferroni's *post-hoc* test. *Scale bar*: 100µm.

Development • Supplementary information

Table S1. The primary and secondary antibodies used in this study.

Primary Antibody	***Host***	***Dilution***	***Catalogue code & Manufacturer***
CALBINDIN	goat pAb	1:500	**sc-7691** - Santa Cruz Biotechnology
CYCLIN D1	rabbit mAb	1:400	**RM-9104** - NeoMarkers (Thermo Scientific)
EPITHELIAL CADHERIN	goat pAb	1:500	**AF748** - R&D Systems
JAG1	rat mAb	1:200	**TS1.15H -** Hybridoma Bank
Ki67	rabbit mAb	1:200	**15580 -** Abcam
LEF1	rabbit mAb	1:400	**2230 -** Cell Signaling Technology
PAX2	rabbit pAb	1:400	**71-6000 -**Invitrogen
PODOCALYXIN	goat pAb	1:300	**AF1556 -** R&D Systems
SIX2	rabbit pAb	1:500	**11562-1-A -** ProteinTech
SOX9	rabbit pAb	1:500	**AB5535 -** Millipore

Secondary Antibody	***Host***	***Dilution***	***Catalogue code & Manufacturer***
anti-goat Alexa Fluor® 488	Donkey	1:400	**705-546-147 -** Jackson Immuno Research Laboratories
anti-rat Cy™3	Donkey	1:400	**712-165-153** - Jackson Immuno Research Laboratories
anti-rabbit Alexa Fluor® 568	Donkey	1:400	**A10042** – ThermoFisher
anti-goat Alexa Fluor® 647	Donkey	1:400	**A-21447 -** ThermoFisher
anti-rabbit IgG (H+L) - Horseradish Peroxidase (HRP) conjugate	Goat	1:2000	**1721034** - Bio-Rad
anti-goat IgG (H+L) - Horseradish Peroxidase (HRP) conjugate	Rabbit	1:500	**1721034 -** Bio-Rad

Table S2. The primers used for qRT-PCR in this study.

GENE	***Forward primer***	***Reverse primer***
Crlf1	GGAATCTGGAGCGAGTGGAG	TATGCGTGCTTCTTGAGCCA
Etv4	CTTTCCATAGCCCCACCACC	GGGGGACTTGATGGCGATTT
Etv5	CAGCCCGCCACGGAG	GCCCTCGACAGTCCTCTGAT
Lama1	TTAGCCATCGGGACCTCAGA	GCAAATGCACATGCCTCCAA
Sostdc1	GGCAGGCATTTCAGTAGCAC	CACTGGCCGTCCGAAATGTA
Srgn	CGTCCTGGTTTGGGGATCTT	TGTGGTCCCTTCTCCTCGAT
Wfdc2	GACAGCCAGTGTTCTGGCAA	CGACTCCAGATGCACAGTCC
Wnt11	ATATCCGGCCTGTGAAGGAC	CACTGCCGTTGGAAGTCTTG
Ctla2a	ACCGTGGACAACAAAATGATGG	TCCAAACTTGGATCAGGGGG
Fgf9	AAAGGGGATTCTCAGGCGG	CGTTCATGCCGAGGTAGAGT
Fgf20	TAAAGGGGATTCTCAGGCGG	TCATGCCGAGGTAGAGTCCA
Wnt4	TGTTCCACACTGGACTCCCT	GTATACAAAGGCCGCCTCCC

Development • Supplementary information

Wnt9b	TGTCAGTTCCAGTTCAGGCA	GGGGAGTCGTCACAAGTACAG
Gdnf	CGCTGACCAGTGACTCCAATATGC	TGCCGCTTGTTTATCTGGTGACC
Gapdh	GCCTCGTCCCGTAGACAAAA	ATGAAGGGGTCGTTGATGGC
Hprt1	CAGTCCCAGCGTCGTGATTA	TGGCCTCCCATCTCCTTCAT
Pgk1	TTGGACAAGCTGGACGTGAA	AACGGACTTGGCTCCATTGT

Development • Supplementary information
