# Supplementary material for: Postnatal prolongation of mammalian nephrogenesis by excess fetal GDNF
Source: Development. 2021 May 25;148(10):dev197475. doi: 10.1242/dev.197475 (PMC8180252; doi:10.1242/dev.197475)
Supplement: Reviewer comments [file develop-148-197475_review_history.pdf]

Postnatal prolongation of mammalian nephrogenesis by excess fetal GDNF

Hao Li, Kristen Kurtzeborn, Jussi Kupari, Yujuan Gui, Edward Siefker, Benson Lu, Kärt Mätlik, Soophie Olfat, Ana R. Montaño-Rodríguez, Sung-Ho Huh, Franklin Costantini, Jaan-Olle Andressoo and Satu Kuure
DOI: 10.1242/dev.197475

Editor: Patrick Tam

Review timeline
Original submission: 1 October 2020
Editorial decision: 4 November 2020
First revision received: 23 March 2021
Accepted: 26 April 2021

Original submission

First decision letter

MS ID#: DEVELOP/2020/197475

MS TITLE: Postnatal prolongation of mammalian nephrogenesis by excess fetal GDNF

AUTHORS: Hao Li, Jussi Kupari, Kristen Kurtzeborn, Yujuan Gui, Edward Siefker, Benson Lu, Kart Matlik, Soophie Olfat, Ana R Montano-Rodriguez, Sung-Ho Huh, Franklin D Costantini, Jaan-Olle Andressoo, and Satu Kuure

I have now received all the referees' reports on the above manuscript, and have reached a decision. The referees' comments are appended below, or you can access them online: please go to BenchPress and click on the 'Manuscripts with Decisions' queue in the Author Area.

As you will see, the referees express considerable interest in your work, but have some significant criticisms and recommend a substantial revision of your manuscript before we can consider publication. If you are able to revise the manuscript along the lines suggested (see also editor's note), which may involve further experiments, I will be happy receive a revised version of the manuscript. Your revised paper will be re-reviewed by one or more of the original referees, and acceptance of your manuscript will depend on your addressing satisfactorily the reviewers' major concerns. Please also note that Development will normally permit only one round of major revision.

We are aware that you may be experiencing disruption to the normal running of your lab that make experimental revisions challenging. If it would be helpful, we encourage you to contact us to discuss your revision in greater detail. Please send us a point-by-point response indicating where you are able to address concerns raised (either experimentally or by changes to the text) and where you will not be able to do so within the normal timeframe of a revision. We will then provide further guidance. Please also note that we are happy to extend revision timeframes as necessary.

Please attend to all of the reviewers' comments and ensure that you clearly highlight all changes made in the revised manuscript. Please avoid using 'Tracked changes' in Word files as these are lost in PDF conversion. I should be grateful if you would also provide a point-by-point response detailing how you have dealt with the points raised by the reviewers in the 'Response to Reviewers' box. If you do not agree with any of their criticisms or suggestions please explain clearly why this is so.

© 2021. Published by The Company of Biologists under the terms of the Creative Commons Attribution License (https://creativecommons.org/licenses/by/4.0/).

Editor's note:

- Appraisal of the impact of renal hypoplasia due to premature cessation of nephrogenesis on the postnatal phenotype in the GDNF mouse model
- lack of quantitative data of nephron progenitors in relation to the reduced fetal nephrogenesis and prolonged (or delayed) postnatal nephrogenesis
- Further information to support the role/ contribution of Wnt11 activity
- Provision of a cohesive view of the gene regulatory circuit/pathway that has been affected, and the impact on the functional attributes of the progenitors by the hyper-GDNF or hyper GDNF/Wnt11 condition

Reviewer 1

Advance summary and potential significance to field

Hao et al. analyze mice with enhanced GDNF activity and demonstrate prolonged nephrogenesis after birth. Nephron progenitors in the postnatal GDNF-hyper mice are maintained longer than the control mice, although high GDNF in the embryonic kidneys results in progenitor depletion. The former finding is novel in the research field of developmental nephrology, and may also be useful for medical strategies to regulate congenital nephron endowment.

Comments for the author

While the overall description of the data is reasonable, the authors underestimate the secondary effects caused by severe renal hypoplasia that occurs before birth. Alternatively, GDNF expression, which is elevated during gestation, may simply return to normal levels after birth, which permits nephrogenesis only postnatally. For GDNF to prolong nephron progenitor lifespan after birth, GDNF would need to be activated specifically after birth. If this is not the case, the authors should tone down their claim throughout the text. In addition, quantitative data showing nephron progenitor maintenance should be included in all of the related figures.

Major points
1. The authors underestimate the secondary effects caused by severe renal hypoplasia that occurs before birth. Inclusion of FGF9/20 knockout data does not completely exclude the possibility of such secondary effects. Alternatively, GDNF expression, which is elevated during gestation may simply return to normal levels after birth, which permits nephrogenesis only postnatally. To unequivocally claim that GDNF prolongs nephron progenitor lifespan after birth, GDNF would need to be activated specifically after birth. The authors should include these points in the discussion and tone down their claim throughout the text, including in the title and abstract.
2. The kidneys of GDNF-hyper mice are hypoplastic at birth and possess smaller numbers of ureteric buds than the control mice. Even if nephrogenesis continues longer, the total number of nephrons that form in such small numbers of nephrogenic niches are likely less than those in the control mice. The authors should present such a calculation and discuss the limitations of this mouse model.
Indeed, the glomerular numbers in the postnatal GDNF-hyper mice are fewer than in the wild-type mice. Thus the previously reported let7/Lin28 manipulation (Yermalovich et al. Nat Commun 2019) is a better model for prolonged postnatal nephrogenesis, and the authors' claims of the superiority of GDNF-hyper mice over other models in terms of tumorgenicity should be toned down.
3. The nephron progenitor numbers should be quantified and statistical analyses should be performed for all of the related figures. The numbers of examined animals and sections/fields per kidney should also be described.
4. Regarding the genetic cross of GDNF-hyper mice with Wnt11+/- mice postnatal nephron progenitor maintenance is not analyzed, although it is the main focus of this manuscript.
Rather, double mutant mice exhibited further renal size reduction before birth. In addition, Wnt11 is not upregulated in the postnatal GDNF-hyper mice. The significance of Wnt11 in postnatal progenitor maintenance is vague in the present form of this manuscript, and it should be explained more clearly.

© 2021. Published by The Company of Biologists under the terms of the Creative Commons Attribution License (https://creativecommons.org/licenses/by/4.0/).

Minor points

1. Explain the principle of GDNF activation in GDNF-hyper mice in the introduction section, as this is a key point that is necessary for the readers to interpret the data precisely.
2. The legend for Supplementary Figure 1F is missing.

Reviewer 2

Advance summary and potential significance to field

There is intense interest in extending nephron progenitor lifespan in the developing kidney because genetic or environmental factors that disrupt progenitor maintenance result in low nephron number and predisposition to kidney disease. The majority of nephrons form just before birth in humans and just after birth in mice. As such, extending this period of nephron formation could have a dramatic impact on nephron number and potentially protect at-risk individuals such as pre-term babies from the detrimental effects of low nephron number.

This work presents a new observation that nephron progenitor cells can be maintained past the point that they would usually differentiate by increased levels of a growth factor important for kidney development- gdnf. However, the work in its current form does not adequately address: the reality that very few progenitors are maintained, the source of gdnf expression, or major morphological abnormalities that are also evident in this model.

Gdnf is an important and well known regulator of kidney development. As such, the field will be interested in this work and take note of the results. However, many would have reservations about this work in its current form.

Comments for the author

This paper presents an analysis of nephron progenitor (NP) cell maintenance and differentiation in a Gdnf hypermorphic mouse line. The finding that NP cells are maintained past the time these cells differentiate in wildtype animals is new, as is the finding that Wnt11 and Gdnf- two genes proposed to interact in a positive feedback loop- have seemingly independent effects on kidney size.

Although of interest, the study is limited by selectively highlighting specific results without due acknowledgment of other phenotypes, or a sense of proportion to the results being described. For example the summary of the article states "growth factor augmentation substantially extends nephron progenitor lifespan and nephrogenesis in postnatal kidney". What this and other sections of the manuscript do not highlight is that the kidney is severely hypoplastic, nephron number is lower than controls, and most nephron progenitors are depleted early in kidney development. However, the few progenitors that remain do so beyond their normal lifespan and continue to generate nephrons. The implication that Gdnf could be used to extend nephrogenesis is intriguing but the effect of higher levels of this growth factor on the rest of the kidney are alarming.

Analysis of proliferation and Gdnf target gene expression led to the proposal that excess Gdnf depletes nephron progenitor cells by suppressing proliferation but maintains these cells for an extended period by increased levels of Wnt11 and other Gdnf targets that are known to influence nephron progenitor maintenance. The link to proliferation is new. While the Wnt11 hypothesis is feasible and consistent with previous work, this finding does not offer additional mechanistic insight into how gdnf or its targets regulate nephron progenitor cells.

A major question arising from this work is where is Gdnf being expressed? The authors focus nephron progenitor cells - “Gdnf, expressed by the nephron progenitor population”, however Magella et al., Dev Biol 2018 identify the cortical stroma as a source of Gdnf, in addition to NP cells. Other single cell data sets support this finding and expression at lower levels in other stromal populations. Given the paucity of NP cells from E14.5 onwards, and the persistent and dramatic effects on the ureteric tip and other areas of the kidney into early postnatal life (see large ureteric tips at P3 in Fig.3), it’s unlikely that NP cells are the major source of Gdnf in this model. Indeed,

© 2021. Published by The Company of Biologists under the terms of the Creative Commons Attribution License (https://creativecommons.org/licenses/by/4.0/).

the authors show a 6-fold increase on wildtype GDNF levels at postnatal day 7, when by their own characterisation the NP population is no longer present.

Suggestions and comments Introduction- Please provide a brief description of the GDNF hyper mouse including the genetic modification, level of expression, and an overview of phenotypes previously associated with that line. Relevant papers are cited but as this mouse forms the basis of the current study it should be described at the start of the manuscript.

The images of E11.5 kidneys presented in Figure 1A show an expanded UB and reduced number of NPs in the Gdnf hyper/hyper mouse compared to control, which are consistent with the culture experiments and E14.5 (Fig1.d,f). The results section reports "NP amount in Gdnfhyper/hyper kidneys resembled that in wild type kidneys but was quickly decreased". Please include a note about the early UB phenotype which is well-illustrated in supp fig1B.

Please include the embryonic stage (E12.5) for this statement as it contrasts with data presented in the same figure from other stages (E14.5). "Quantification of NP cell amounts confirmed similar NP amounts in both genotypes, while analysis of pHH3 specifically in NP population revealed 57% reduction in mitotic cells of (Figure 1G-H)." Please provide more detail about how these samples were imaged and how cell numbers were quantified. The description in the methods was not detailed enough to understand the experiment. Were any other stages quantified?

Regarding the in vitro experiment assessing "whether recombinant GDNF can control NP cell proliferation in vitro", were multiple concentrations tested? Was the 100ng/mL concentration based on any prior work or optimisation? If so, please include a reference or comment to this effect.

Please consider expanding or clarifying the statement "The results show general decrease in early nephron precursor numbers and indicate nephron differentiation hysteresis in embryonic Gdnf hyper/hyper kidneys". Assuming hysteresis means the history of dependence- what is the dependence and altered response in reference?

The figure legend description is missing for supplementary figure 1F. Please provide some representative images for this analysis. This figure describes glomerular density in mm^2, a proxy for glomerular/nephron number, with text highlighting lower overall density in gdnf hyper mice, but an increase in proportional glomerular density in the cortex in comparison to controls. The conclusions and comparisons from this analysis may be confounded by other aspects of the phenotype such as changes in kidney size, the noted decrease in nephron formation in Gdnf hyper mice earlier in development, and the large cysts reported to form at this stage (Kumar et al., 2015). Please include some text in the supplementary figure or methods detailing how these factors were accounted for or perhaps focus on the results that despite a reduced NP population, nephron formation continues?

The following statement referencing the proportional differences in glomerular density should be revised: "This shows that despite the early deceleration of NP self-renewal, a larger proportion of late born nephrons develop in the kidneys facing excess GDNF during organogenesis." At present it is unlikely to be true as Short et al., 2014 showed that over 50% of nephrons form after birth during normal development. "late born nephrons" would encompass that 50% of nephrons born after birth in the control so without measuring the number of nephrons before birth and into adulthood this is still unclear. The number of nephrons would be much lower in hyper/hyper mice.

Regarding the retention of some NP cells after cessation- the data in Figure 3 shows a number of examples where a thin layer of NP cells is present in gdnf hyper/hyper kidneys and not controls. While the Gdnf hyper/hyper mice are clearly delayed in their cessation of nephrogenesis, the staining referenced in Fig4C is more in line with an early committing nephron than persistent NP cells at this stage. Rumballe et al., Dev Biol., 2011, report the transition from NP -> early committing nephron at cessation is marked partly by a change in localisation of NP cells, but primarily by the formation of a Collagen IV+ basement membrane around NP clusters, which coincides with morphological evidence of increasing polarity - seen as a rosette of NP nuclei with a gap in the centre (Rumballe et al., Fig 3b and c). These signs of polarisation are evident in Fig. 4C (arrow on the left), the central arrow points to an isolated cell, the right arrow points to a cluster

© 2021. Published by The Company of Biologists under the terms of the Creative Commons Attribution License (https://creativecommons.org/licenses/by/4.0/).

of cells similar to one marked in the control (asterisk in the right of Fig. 4A). This section could perhaps focus on a consistent delay in gdnf hyper/hyper mice compared to controls.

Authors point out how an excess of GDNF can prolong the nephrogenic program and compare this to other models like Lin28/Let7 overexpression which create a much more dramatic phenotype, but can lead to cancer. However, they fail to acknowledge the dramatic abnormalities associated with the Gdnf hyper/hyper mice.

The authors comment on the phenotype of mice with a single Gdnf hyper allele stating that this single allele is insufficient to maintain nephron progenitor cells however they only show data from postnatal day 5 (supp fig 5 E,F), which past the point that NP cells are maintained in the gdnf hyper/hyper line.

Figure 6: are new nephrons formed at P7 or are these the same nephrons that formed at P4? Are these nephrons stalled at an early stage?

The paper focuses on nephron progenitor maintenance and extension of nephrogenesis but NP cells are only visualised early in kidney development (E11.5, 14.5) and at postnatal day 3. It would be useful to know whether this population remains scarce, or has a resurgence between E15.5-P2.

Comment- Figure 3 D seems to show a strong upregulation of Pax2 in the ureteric tip compared to controls.

Reviewer 3

Advance summary and potential significance to field

In "Postnatal prolongation of mammalian nephrogenesis by excess fetal GDNF" Hao and colleagues describe the nephron progenitor phenotype of Gdnf hyper mice during the first week of postnatal development.

The authors describe that Â“excess Gdnf, expands the nephrogenic program beyond its normal cessation by maintaining NP cells and nephrogenesis in postnatal mouse kidneysÂ”

There is an ongoing effort to understand how nephron progenitors can be expanded in vitro for experimental purposes and
potential regenerative therapeutic ends, and with a growing number of premature births surviving, it is important to recognize the impact of premature cessation of nephrogenesis and the impact this has on nephron endowment and ultimately quality of life and health. Further, there is an inherent interest in understanding the biology underpinning nephron progenitor growth, expansion and differentiation.

The significance of this manuscript rests on its finding that higher expression of Gdnf can result in a delayed end to nephrogenesis. The delay is from approximately day 4 to day 7 of postnatal development. The debate of whether nephron progenitors commit to differentiate during this period due to an intrinsic or extrinsic clock, or a change in environment, is still on going.

The authors show the following:
1. in Gdnf-hyper mice NPCs persist longer.
2. Fgf9/Fgf20 deficient mice do not display this (argument being hypoplasia is therefore not cause of NPC survival in Gdnf-hyper mice)
3. The Gdnf-hyper mice phenotype can be modified on a Wnt11 het background.

As a reviewer, what I am missing is the obvious connection between these experiments, and some form of cohesive view of a gene regulatory circuit/pathway being modified. How is the biology of NPCs altered on the hyper or hyper/Wnt11 backgrounds? Without these, it is not clear that this warrants publication in Development at this time as the advance remains primarily descriptive.

© 2021. Published by The Company of Biologists under the terms of the Creative Commons Attribution License (https://creativecommons.org/licenses/by/4.0/).

Comments for the author

The authors build up an argument that initial NPC endowment is similar in wildtype and Gdnf-hyper kidneys but this relies on data in Figure 1/S1 and it is not clear how NPCs are quantified, is it per area, per kidney, per tip?

The authors do state the analyses are done on E12.5 kidneys, but if the Gdnf-hyper kidneys have altered branching morphogenesis and tip numbers, are they taking this into account? The authors quantify NPC amounts at E12.5 and argue that the start-point are similar for wt and hyper kidneys. However, in Figure 1A-B there is a clear difference at E11.5 (which is not quantified), and similarly, there is a striking difference at E14.5 (also not quantified) which begs the question of whether the E12.5 data is indeed accurate. Better descriptions of the quantification and the caveats of the quantification method are needed.

The authors also quantify PHH3+ NPCs in wildtype and hyper kidneys as well as in cultured kidneys exposed to Gdnf and show a consistent decrease, concomitant with reduced nephrogenesis. Again, more detailed descriptions of how this was performed and the caveats (as based on the other Gdnf phenotypes exhibited) are needed.

The authors describe cortical to cortical-to-total glomerular density as a measurement where wildtype and hyper kidneys differ. This raises the question, are all renal corpuscles not typically positioned in the cortex? The is a fairly sharp boundary between the Outer Medulla and the Cortex where renal corpuscle, S1, and S2 are within the Cortex. Could the authors elaborate on the meaning of this wording and how the analyses were performed?

"However, our analysis failed to detect SIX2- and/or PAX2-positive cells in the NP niche of Fgf9;20 deficient kidneys (Supplemental figure 2). This together with published data (O'Brien, 2018; Urbach et al., 2014; Yermalovich et al., 2019)
suggests that prolonged nephrogenesis derives from increased GDNF, not from renal hypoplasia." While this is possible, it could also be due to renal hypoplasia. I would argue a negative outcome here i.e. non-detection of Six2 NPCs in Fgf9/Fgf20 deficient kidneys, does not suggest a positive conclusion. Renal hypoplasia can occur due to many conflicting situations. All the authors can conclude is that the phenotype in Fgf9/Fgf20 deficient mice is not the same as in the Gdnf-hyper kidneys. Without the underlying mechanism it is hard to make further arguments.

"Removal of one functional Wnt11 allele in Gdnfhyper/hyper background (Gdnfhyper/hyper;Wnt1+/-) did not improve postnatal kidney size from that in Gdnfhyper/hyper pups (Supplemental figure 7A-E). However, removal of Wnt11 allele in Gdnfhyper/hyper individuals enhanced SIX2-positive NP population and improved nephrogenesis, as evidenced by increased PAX2-positivity in the cortical differentiation zone of Gdnfhyper/hyper;Wnt1+/- kidneys (Figure 8, compare also to Figure 1F). Thus, this result suggests that WNT11 mediates GDNF's regulatory functions to NP cells."

There is a typo in the above sentence but more importantly this is not supported by clear quantification of Six2+ progenitor populations.

Further, knowing that modifications to Gdnf signalling alters branching morphogenesis, is it not plausible that a higher level of Gdnf protein results in expanded tips, with consequent larger Wnt11 expression domains? The Gdnfhyper/hyper; Wnt11+/- could therefore just have restored the tip size? The authors describe this phenotype in S7 (red arrows). Following on from that the authors describe "cyst in glomerular tuft (black arrow)" in S7D-E. The glomerular tuft refers to the capillaries that form the glomerulus. To me it looks like these cysts are made from an expansion of the Bowman's space.

"Gdnf, expressed by the nephron progenitor population, is lost from wild type kidneys by P2 (www.gudmap.org) while its mRNA and protein were still present in Gdnfhyper/hyper kidneys as late as P7 (Figure 7A-D)."

© 2021. Published by The Company of Biologists under the terms of the Creative Commons Attribution License (https://creativecommons.org/licenses/by/4.0/).

Given the importance of understanding the dynamics of Gdnf expression during postnatal days of development, it is surprising to me that the authors did not perform a time-course of in situ hybridization experiments to pinpoint the exact differences between wildtype and hyper kidneys.

I was under the impression that mouse genes are written as follows Gdnf while their protein are Gdnf. This contrasts to human nomenclature GDNF/GDNF.

Supplemental figure 1 lacks texts describing S1F.

First revision

Author response to reviewers' comments

Reviewer 1 *Advance Summary and Potential Significance to Field:*
Hao et al. analyze mice with enhanced GDNF activity and demonstrate prolonged nephrogenesis after birth. Nephron progenitors in the postnatal GDNF-hyper mice are maintained longer than the control mice, although high GDNF in the embryonic kidneys results in progenitor depletion. The former finding is novel in the research field of developmental nephrology, and may also be useful for medical strategies to regulate congenital nephron endowment.

Comments for the Author:
While the overall description of the data is reasonable, the authors underestimate the secondary effects caused by severe renal hypoplasia that occurs before birth. Alternatively, GDNF expression, which is elevated during gestation, may simply return to normal levels after birth, which permits nephrogenesis only postnatally. For GDNF to prolong nephron progenitor lifespan after birth, GDNF would need to be activated specifically after birth. If this is not the case, the authors should tone down their claim throughout the text. In addition, quantitative data showing nephron progenitor maintenance should be included in all of the related figures.

Response: We thank the reviewer for acknowledging the importance of our results and pointing out our overtly positive way of describing and interpreting them. We appreciate valuable and knowledgeable comments and have now addressed them substantially in the revised manuscript (edits and new results written in red).

Please see our responses to the specific comments below.

Major points
1. The authors underestimate the secondary effects caused by severe renal hypoplasia that occurs before birth. Inclusion of FGF9/20 knockout data does not completely exclude the possibility of such secondary effects. Alternatively, GDNF expression, which is elevated during gestation, may simply return to normal levels after birth, which permits nephrogenesis only postnatally. To unequivocally claim that GDNF prolongs nephron progenitor lifespan after birth, GDNF would need to be activated specifically after birth. The authors should include these points in the discussion and tone down claim throughout the text, including in the title and abstract.

Response: This is very important point that, as the reviewer acknowledges, we had already addressed in our original submission, though we agree we cannot draw definite conclusion without activating endogenous GDNF expression after birth.
We agree with the reviewer about the dynamics of the elevated GDNF expression, and in the abstract of our initial manuscript we tried to communicate this by stating "*Decline of GDNF levels in postnatal kidneys normalizes the ureteric bud niche and creates a permissive environment for continuation of prolonged nephrogenic program, as demonstrated by morphologically and molecularly normal postnatal nephron progenitor self-renewal and differentiation.*" In the revised manuscript we have modified this to better highlight this possibility.
The impact of renal hypoplasia was originally addressed in FGF9;20 mutant kidneys, and thorough literature search. We agree that although neither of these indicate that small size (=hypoplasia) per

© 2021. Published by The Company of Biologists under the terms of the Creative Commons Attribution License (https://creativecommons.org/licenses/by/4.0/).

se would be able to maintain nephron progenitors longer than in "normal sized" kidney, they do not comprehensively exclude that possibility.
Recently several new studies demonstrate wide variability in healthy kidney size and timing of nephrogenesis cessation (Bennett et al., 2020; Bonsib, 2020; Charlton et al., 2020; Kuure and Sariola, 2020; Ryan et al., 2018; Sutherland et al., 2011). None of these shows or suggests correlation between reduced kidney size and persistence of nephrogenesis, which should manifest as small kidneys where glomerular density is higher than in normal sized kidneys.

We have now additionally also analyzed the postnatal presence of nephron progenitors in a model where renal hypoplasia is caused by impaired ureteric bud branching (similarly as in *Gdnf*$^{hyper/hyper}$ mice), namely *Hoxb7Cre; Mek1;Mek2* mice. Deletion of all four alleles results in very severe renal hypodysplasia and newborn lethality (Ihermann-Hella et al., 2014) but inactivation of ¾ alleles is compatible with life as kidneys show only mildly reduced size. Again, no signs of prolonged nephrogenesis could be detected (Fig. S3), strongly supporting the view that renal hypoplasia as such is not enough to maintain postnatal nephrogenesis. However, we do recognize that other renal defects in *Gdnf*$^{hyper/hyper}$ mice evoke severe concerns that deserve attention and have now substantially discussed about these in the revised manuscript and toned down claims about GDNF's positive effects in abstract, end of introduction and discussion chapters.
Based on these and other results and views we present in the revised manuscript we hope that our original title still faithfully reflects our findings as this is what actually we show to happen; fetal increased GDNF expression has a major impact on nephron progenitors.

2. The kidneys of GDNF-hyper mice are hypoplastic at birth and possess smaller numbers of ureteric buds than the control mice. Even if nephrogenesis continues longer, the total number of nephrons that form in such small numbers of nephrogenic niches are likely less than those in the control mice. The authors should present such a calculation and discuss the limitations of this mouse model. Indeed, the glomerular numbers in the postnatal GDNF-hyper mice are fewer than in the wild-type mice. Thus, the previously reported let7/Lin28 manipulation (Yermalovich et al. Nat Commun 2019) is a better model for prolonged postnatal nephrogenesis, and the authors' claims of the superiority of GDNF-hyper mice over other models in terms of tumorgenicity should be toned down.

Response: We agree and apologize for not bringing up enough the branching morphogenesis defect of *Gdnf*$^{hyper/hyper}$ mice that we have published earlier (Li et al., 2019) and which clearly results in hypodysplastic kidneys with reduced nephron endowment despite the continued postnatal nephrogenesis.

In the revised manuscript we have now described the glomerular density results in a manner that will give the reader more thorough understanding of the effects that fetal excess of GDNF has on renal differentiation in general and especially on glomerular density, which is indeed not improved to the level of wild type kidneys (Table 1 of the revised manuscript). We have also removed the superiority statements of GDNF-hyper mice over other models throughout the text.

3. The nephron progenitor numbers should be quantified and statistical analyses should be performed for all of the related figures. The numbers of examined animals and sections/fields per kidney should also be described.

Response: The overall nephron progenitor numbers were quantified in the original manuscript at the initiation of branching morphogenesis (E12.5) in *Gdnf*$^{hyper/hyper}$ kidneys.
In the revised manuscript the overall nephron progenitor numbers are additionally quantified at E11.5 and E14.5 kidneys. These show "*an initial increase in NPC numbers, which was transiently normalized at E12.5 but severely decreased at E14.5 Gdnf*$^{hyper/hyper}$ *kidneys (Fig. 1G-H, S1E-F)*".
Comparison of the nephron progenitor numbers in *Gdnf*$^{hyper/hyper}$ and *Gdnf*$^{hyper/hyper}$; *Wnt11*$^{+/-}$ at E14.5 and P0 revealed that removal of one *Wnt11* allele resulted in 1,4 and 1,5 higher average of nephron progenitors/niche, respectively (Fig. 8 of revised manuscript).
We have also quantified how many of nephrogenic niches have nephron progenitors in wild type and *Gdnf*$^{hyper/hyper}$ kidneys at P3-P6 (Fig3-4, S1H). This revealed that while in wild type animals at P3 10% of the UB tips (niches) are capped with NPCs (Fig. 3A, 48 tips analyzed in 2 kidneys), in *Gdnf*$^{hyper/hyper}$ mice 59% of analyzed niches have NPs at the same age (Fig. 3B, 107 tips analyzed in 2 kidneys). At P4 and P5 NPCs were undetectable in wild type niches (Fig. 4A-B, 315 and 10 tips

© 2021. Published by The Company of Biologists under the terms of the Creative Commons Attribution License (https://creativecommons.org/licenses/by/4.0/).

analyzed, respectively). At P6 calbindin-positive epithelium resembling tip morphology was almost undetectable in the wild type kidney cortex, which restricted substantially the number of tips that could be analyzed. In P4 *Gdnf*$^{hyper/hyper}$ kidneys NPCs were detected in 18% of the analyzed niches while only committed NPCs were detected at P6 (Fig. 4C-D, 245 and 16 tips analyzed, respectively).

4. Regarding the genetic cross of GDNF-hyper mice with Wnt11+/- mice, postnatal nephron progenitor maintenance is not analyzed, although it is the main focus of this manuscript. Rather, double mutant mice exhibited further renal size reduction before birth. In addition, Wnt11 is not upregulated in the postnatal GDNF-hyper mice. The significance of Wnt11 in postnatal progenitor maintenance is vague in the present form of this manuscript, and it should be explained more clearly.

Response: We thank the reviewer for the constructive comment on the complex outcome of this genetic cross and agree with challenges in the interpretation of the results.
We would like to start by pointing out that the size reduction seen in newborn *Gdnf*$^{hyper/hyper}$ kidneys after deletion of one *Wnt11* allele likely reflects improved morphology of the *Gdnf*$^{hyper/hyper}$; *Wnt11*$^{+/-}$ kidneys in comparison to *Gdnf*$^{hyper/hyper}$ kidneys (please see revised manuscript fig. 8E-H, S9D-E). *Gdnf*$^{hyper/hyper}$ kidneys typically have big collecting duct cysts leading to severely enlarged pelvis, which is not seen in four analyzed *Gdnf*$^{hyper/hyper}$; *Wnt11*$^{+/-}$ kidneys. This result shows, as we have stated in the revised manuscript, that lowering *Wnt11* dosage in *Gdnf*$^{hyper/hyper}$ background fails to rescue kidney size but it also suggests that *Wnt11* reduction diminishes ureteric bud derived defects thus resulting in smaller kidney size.
Next, we have tried to generate new pups to complement the nephron progenitor analysis in *Gdnf*$^{hyper/hyper}$; *Wnt11*$^{+/-}$ beyond the newborn stage. Unfortunately we failed to generate any *Gdnf*$^{hyper/hyper}$; *Wnt11*$^{+/-}$ offspring in the timeframe of manuscript revision and could therefore not provide additional data on this. We hope that the reviewer appreciates our other efforts to address this question as described above (=improved NPC/niche in *Gdnf*$^{hyper/hyper}$; *Wnt11*$^{+/-}$) and as further detailed below.
To gain more information on the mechanisms how GDNF can regulate nephron progenitors we performed additional experiments to identify gene regulatory networks possibly affected by excess GDNF, which produces huge ureteric bud. We did this by analyzing Wnt/b-cateinin targets, which could mediate increased canonical WNT signaling as response to upregulated *Wnt9b* and *-11* in ureteric buds (Fig. 7), and previously shown to negatively impact nephron progenitor maintenance (Dapkunas et al., 2019; Kiefer et al., 2012). This analysis detected significant increase in *axin2* expression at E14.5 *Gdnf*$^{hyper/hyper}$ kidneys (Fig. S7A in revised manuscript). Notably, the nephron progenitor specific Wnt/b-catenin targets *Amph, Cited1* and *Uncx4.1* (Karner et al., 2011) showed trend of downregulation, likely reflecting the overall decrease in nephron progenitor numbers in embryonic *Gdnf*$^{hyper/hyper}$ kidneys. In this light the slightly increased but statistically insignificant *Tafa4* expression is interesting and suggests that increased canonical WNT signaling contributes to observed inhibitory function of excess GDNF on NPCs. This is supported by our new experiments, which show that chemical inhibition of WNT signaling in the presence of excess GDNF significantly alleviates extended ureteric bud tip morphology (Fig. S8). Of note, WNT inhibition by IWR1 and IWP2 (data not shown) disrupts nephron progenitor coherence, which likely derives from the imbalance the chemicals cause to progenitor autonomous WNT signaling.
The expression of canonical WNT targets is reversed in P5 *Gdnf*$^{hyper/hyper}$ kidneys, where *Wnt9b* and *-11* no longer are significantly upregulated (Fig. 7F). Our new results however show that their canonical targets *Cited1* and *Uncx4.1* (Karner et al., 2011) together with *R-spondin1*, a known Wnt signaling agonist, which together *R-spondin3* is essential for the nephron progenitor maintenance and differentiation (de Lau et al., 2014; Vidal et al., 2020), showed increased expression in *Gdnf*$^{hyper/hyper}$ kidneys (Fig. S7B).

These new results together with our data in Fig. 7 suggest that increased GDNF levels augment several bud- derived GDNF/Ret targets, which not alone but in combination with each other and together with increased canonical WNT signaling are inhibitory to nephron progenitor proliferation and differentiation in embryonic kidneys. In postnatal kidneys, GDNF expression gradually decreases and generates permissive environment for normal nephrogenesis. The results also suggest that GDNF may act as a previously unrecognized master regulator of ureteric bud-derived signals that function to control events in the nephrogenic niche.

© 2021. Published by The Company of Biologists under the terms of the Creative Commons Attribution License (https://creativecommons.org/licenses/by/4.0/).

Minor points

1. Explain the principle of GDNF activation in GDNF-hyper mice in the introduction section, as this is a key point that is necessary for the readers to interpret the data precisely.

Response: We have now added an explanation for genetic upregulation of GDNF to the Introduction section of the revised manuscript (p. 4, last chapter).

2. The legend for Supplementary Figure 1F is missing.

Response: We apologize for the missing legend. In the revised manuscript Fig. S1F is designated as Table 1 and the legends is provided.

Reviewer 2 *Advance Summary and Potential Significance to Field:*
There is intense interest in extending nephron progenitor lifespan in the developing kidney because genetic or environmental factors that disrupt progenitor maintenance result in low nephron number and predisposition to kidney disease. The majority of nephrons form just before birth in humans and just after birth in mice. As such, extending this period of nephron formation could have a dramatic impact on nephron number and potentially protect at-risk individuals such as pre-term babies from the detrimental effects of low nephron number.
This work presents a new observation that nephron progenitor cells can be maintained past the point that they would usually differentiate by increased levels of a growth factor important for kidney development- gdnf. However, the work in its current form does not adequately address: the reality that very few progenitors are maintained, the source of gdnf expression, or major morphological abnormalities that are also evident in this model.
Gdnf is an important and well known regulator of kidney development. As such, the field will be interested in this work and take note of the results. However, many would have reservations about this work in its current form.

Response: We thank the reviewer for acknowledging the importance of our results and pointing out the shortcomings of our original manuscript. We appreciate valuable and knowledgeable comments and have now addressed them substantially in the revised manuscript (edits and new results written in red).

Please see our responses to the specific comments below.

Comments for the Author:
1. This paper presents an analysis of nephron progenitor (NP) cell maintenance and differentiation in a Gdnf hypermorphic mouse line. The finding that NP cells are maintained past the time these cells differentiate in wildtype animals is new, as is the finding that Wnt11 and Gdnf- two genes proposed to interact in a positive feedback loop- have seemingly independent effects on kidney size.

Although of interest, the study is limited by selectively highlighting specific results without due acknowledgment of other phenotypes, or a sense of proportion to the results being described. For example the summary of the article states "growth factor augmentation substantially extends nephron progenitor lifespan and nephrogenesis in postnatal kidney". What this and other sections of the manuscript do not highlight is that the kidney is severely hypoplastic, nephron number is lower than controls, and most nephron progenitors are depleted early in kidney development. However, the few progenitors that remain do so beyond their normal lifespan and continue to generate nephrons. The implication that Gdnf could be used to extend nephrogenesis is intriguing but the effect of higher levels of this growth factor on the rest of the kidney are alarming.

Response: We thank the reviewer for remarking our clearly too overtly positive interpretation of the results. We agree that our earlier work which shows branching defect and hypodysplastic kidneys in *Gdnf*$^{hyper/hyper}$ mice (Li et al., 2019) should be better explained to properly contextualize our results.
In the revised manuscript we have now pointed these out in several different sections. We have additionally described the glomerular density results in a manner that will give the reader more

© 2021. Published by The Company of Biologists under the terms of the Creative Commons Attribution License (https://creativecommons.org/licenses/by/4.0/).

thorough understanding of effects that the fetal excess of GDNF has on glomerular density, which is not improved to the level of wild type kidneys (Table 1 of the revised manuscript).
We hope that the reviewer appreciates our attempt to tune down overtly positive interpretation of the results throughout the revised manuscript. Changes are shown in red font throughout the text. These are exemplified by the change of Summary statement, which now runs: "*We show that the dosage of neurotropic factor GDNF regulates of nephron progenitors and that in utero growth factor augmentation can extend postnatal lifespan and differentiation of nephron progenitors*."
We have also revised the last sentence of Discussion, which referred to the use of GDNF as a clinical mean to extend nephrogenesis: "*Additional experimentation is needed to identify possible means to excessively activate GDNF signaling without harmful effects on kidney growth*."

2. Analysis of proliferation and Gdnf target gene expression led to the proposal that excess Gdnf depletes nephron progenitor cells by suppressing proliferation but maintains these cells for an extended period by increased levels of Wnt11 and other Gdnf targets that are known to influence nephron progenitor maintenance. The link to proliferation is new. While the Wnt11 hypothesis is feasible and consistent with previous work, this finding does not offer additional mechanistic insight into how gdnf or its targets regulate nephron progenitor cells.

Response: We acknowledge the reviewer for pointing out insufficient analysis of nephron progenitor regulation in our initial manuscript.
To gain more information on the mechanisms how GDNF regulates nephron progenitors we have now performed additional experiments to identify gene regulatory networks possibly affected by excess GDNF and enlarged ureteric bud. Based on previous information showing that increased canonical WNT signaling negatively impacts nephron progenitor maintenance (Dapkunas et al., 2019; Kiefer et al., 2012) we first analyzed the expression of Wnt/b-cateinin targets, which could mediate the effects of upregulated *Wnt9b* and *-11* in *Gdnf*$^{hyper/hyper}$ ureteric buds reported in our initial manuscript (Fig. 7). This analysis detected significant increase in *axin2* expression in *Gdnf*$^{hyper/hyper}$ kidneys at E14.5 (revised Fig. S7A). Notably, the nephron progenitor specific Wnt/b-catenin targets *Amph*, *Cited1* and *Uncx4.1* (Karner et al., 2011) showed trend of downregulation likely reflecting the overall decrease in nephron progenitor numbers. In this light the slightly increased but statistically insignificant *Tafa4* expression is interesting and suggests that increased canonical WNT signaling contributes to observed inhibitory function of excess GDNF on NPCs.
This is supported by our new experiments, which show that chemical inhibition of WNT signaling in the presence of excess GDNF alleviates extended ureteric bud tip morphology (Fig. S8). Of note, WNT inhibition by IWR1 and IWP2 (data not shown) disrupts nephron progenitor coherence, which likely derives from the imbalance the chemicals cause to progenitor autonomous WNT signaling.
The expression of canonical WNT targets is reversed in P5 *Gdnf*$^{hyper/hyper}$ kidneys, where *Wnt9b* and *-11* no longer are significantly upregulated (Fig. 7F). Our new results however show that their canonical targets *Cited1* and *Uncx4.1* (Karner et al., 2011) together with *R-spondin1*, a known Wnt signaling agonist, which together *R-spondin3* is essential for the nephron progenitor maintenance and differentiation (de Lau et al., 2014; Vidal et al., 2020), showed increased expression in *Gdnf*$^{hyper/hyper}$ kidneys (Fig. S7B).
These new results together with our data in Fig. 7 suggest that increased GDNF levels augment several bud- derived GDNF/Ret targets, which not alone but in combination with each other and together with increased canonical WNT signaling are inhibitory to nephron progenitor proliferation and differentiation in embryonic kidneys. In postnatal kidneys, GDNF expression gradually decreases and generates permissive environment for normal nephrogenesis. The results also suggest that GDNF may act as a previously unrecognized master regulator of ureteric bud-derived signals that function to control events in the nephrogenic niche.

3. A major question arising from this work is where is Gdnf being expressed? The authors focus nephron progenitor cells - "Gdnf, expressed by the nephron progenitor population", however Magella et al., Dev Biol 2018 identify the cortical stroma as a source of Gdnf, in addition to NP cells. Other single cell data sets support this finding and expression at lower levels in other stromal populations. Given the paucity of NP cells from E14.5 onwards, and the persistent and dramatic effects on the ureteric tip and other areas of the kidney into early postnatal life (see large ureteric tips at P3 in Fig.3), it's unlikely that NP cells are the major source of Gdnf in this model. Indeed, the authors show a 6-fold increase on wildtype GDNF levels at postnatal day 7, when by their own characterisation the NP population is no longer present.

© 2021. Published by The Company of Biologists under the terms of the Creative Commons Attribution License (https://creativecommons.org/licenses/by/4.0/).

Response: As the reviewer points out, the single cell sequencing data indicates that GDNF is expressed also in renal stroma. We have performed *in situ* hybridization analysis of *Gdnf* expression throughout development to compare its localization in wild type and *Gdnf*$^{hyper/hyper}$ kidneys (figure below). This reveals increased expression without major changes in the transcript localization pattern, reflecting the result shown in main figure 7 of manuscript.
We have now rephrased the sentences referring to *Gdnf* expressing cells in the revised manuscript (p. 3 and 8 of the revised manuscript). In the introduction, we added metanephric mesenchyme as a source of *Gdnf* and in the beginning of the Results chapter "Persistent postnatal nephrogenesis depends on changes in GDNF induced signaling") the new sentence now runs "*Gdnf expression is lost from wild type kidneys by P2 (www.gudmap.org) while its mRNA and protein were still present in Gdnfhyper/hyper kidneys as late as P7 (Fig. 7A-D).*"

We have removed unpublished data provided for the referees in confidence.

Suggestions and comments

1. Introduction- Please provide a brief description of the GDNF hyper mouse including the genetic modification, level of expression, and an overview of phenotypes previously associated with that line. Relevant papers are cited but as this mouse forms the basis of the current study it should be described at the start of the manuscript.

Response: We agree that this is important and have now added this information to the Introduction section of the revised manuscript (p. 4, last chapter). The sentence now runs: "*We previously reported that genetic disruption of Gdnf's 3' untranslated region causes 3-6-fold increased expression of endogenous GDNF and results in renal hypoplasia due to the UB branching defect (Kumar et al., 2015).*"

2. The images of E11.5 kidneys presented in Figure 1A show an expanded UB and reduced number of NPs in the Gdnf hyper/hyper mouse compared to control, which are consistent with the culture experiments and E14.5 (Fig1.d,f). The results section reports "NP amount in Gdnfhyper/hyper kidneys resembled that in wild type kidneys, but was quickly decreased". Please include a note about the early UB phenotype, which is well-illustrated in supp fig1B. Please include the embryonic stage (E12.5) for this statement as it contrasts with data presented in the same figure from other stages (E14.5). "Quantification of NP cell amounts confirmed similar NP amounts in both genotypes, while analysis of pHH3 specifically in NP population revealed 57% reduction in mitotic cells of (Figure 1G-H)." Please provide more detail about how these samples were imaged and how cell numbers were quantified. The description in the methods was not detailed enough to understand the experiment. Were any other stages quantified?

Response: We have now added stages to our analysis (E11.5, E12.5, E14.5 and postnatal days 0, 3-6) and clarified their description. We have also included abnormal UB to the sentence describing nephron progenitors in *Gdnf*$^{hyper/hyper}$ kidneys in beginning of the chapter "The embryonic nephrogenic program depends on GDNF" on p. 4. The sentence now runs: "*NPs in Gdnf*$^{hyper/hyper}$ *kidneys were distributed around abnormally wide UB as a thinner layer (Fig. 1A-F, S1A-B).*"
We have now also described in more detail the imaging and quantification processes in the Methods section of revised manuscript.

In addition to quantification of the nephron progenitor numbers at E12.5 in the original manuscript, we have now additionally quantified at E11.5 and E14.5 kidneys and included the result: "*Quantification of NPs at E11.5 revealed an initial increase in NPC numbers, which was transiently normalized at E12.5 but severely decreased at E14.5 Gdnf*$^{hyper/hyper}$ *kidneys (Fig. 1G-H, S1E-F)*".

Comparison of the nephron progenitor numbers in *Gdnf*$^{hyper/hyper}$ and *Gdnf*$^{hyper/hyper}$; *Wnt11*$^{+/-}$ at E14.5 and P0 revealed that removal of one *Wnt11* allele resulted in 1,4 and 1,5 higher average of nephron progenitors/niche, respectively (Fig. 8 of revised manuscript).
In addition, we have now quantified how many of nephrogenic niches have nephron progenitors in wild type and *Gdnf*$^{hyper/hyper}$ kidneys at P3-P6 (Fig3-4, S1H). This revealed that while in wild types only 10% of the analyzed UB tips (niches) were capped with NPCs (Fig. 3A, 48 tips analyzed in 2 kidneys) at P3, 59% of analyzed niches in *Gdnf*$^{hyper/hyper}$ have NPs at P3 (Fig. 3B, 107 tips analyzed in 2 kidneys). At P4 and P5 NPCs were not detected in wild type niches (Fig. 4A-B, 315 and 10 tips analyzed, respectively). Of note, it was really difficult to find calbindin-positive epithelium resembling tip morphology in the cortex of P6 wild type kidneys, which restricted the analyzed tip

© 2021. Published by The Company of Biologists under the terms of the Creative Commons Attribution License (https://creativecommons.org/licenses/by/4.0/).

numbers substantially. In P4 *Gdnf*$^{hyper/hyper}$ kidneys NPCs were detected in 18% of the analyzed niches while only committed NPCs were detected at P6 (Fig. 4C-D, 245 and 16 tips analyzed, respectively).

3. Regarding the in vitro experiment assessing "whether recombinant GDNF can control NP cell proliferation in vitro", were multiple concentrations tested? Was the 100ng/mL concentration based on any prior work or optimisation? If so, please include a reference or comment to this effect.

Response: We apologize for not including reference for the concentration of GDNF protein in the initial manuscript. The reference to the original paper (Sainio et al., 1997) is now included in the revised manuscript (p. 14, chapter "Organ culture"). Of note, GDNF is used at 50-100ng/ml concentrations in many other publications, few of which are referred here (Kuure et al., 2005; Lu et al., 2009; Menshykau et al., 2019; Ola et al., 2011).

4. Please consider expanding or clarifying the statement "The results show general decrease in early nephron precursor numbers and indicate nephron differentiation hysteresis in embryonic Gdnf hyper/hyper kidneys". Assuming hysteresis means the history of dependence- what is the dependence and altered response in reference?

Response: Thank you for the note, we have now replaced "hysteresis" with reduced nephron differentiation. The sentence now runs: "*This showed general decline in early nephron precursor numbers and indicate reduced nephron differentiation in embryonic Gdnf*$^{hyper/hyper}$ *kidneys (Figure 2)*"

5. The figure legend description is missing for supplementary figure 1F. Please provide some representative images for this analysis. This figure describes glomerular density in mm^2, a proxy for glomerular/nephron number, with text highlighting lower overall density in gdnf hyper mice, but an increase in proportional glomerular density in the cortex in comparison to controls. The conclusions and comparisons from this analysis may be confounded by other aspects of the phenotype such as changes in kidney size, the noted decrease in nephron formation in Gdnf hyper mice earlier in development, and the large cysts reported to form at this stage (Kumar et al., 2015). Please include some text in the supplementary figure or methods detailing how these factors were accounted for or perhaps focus on the results that despite a reduced NP population, nephron formation continues?

Response: We apologize for the missing legend. The revised manuscript includes legend for the Fig. S1F, which is now designated as Table 1. In the revised manuscript, the representative images are shown in Fig. S5A-B.
The glomerular density measurements as such take into account the overall area of the kidney, where the number of glomeruli are counted, but indeed fails to take into account the size increase caused by large cysts. We followed the reviewer's suggestion to focus on the description of these results to emphasize continued nephrogenesis in *Gdnf*$^{hyper/hyper}$ (p.5, please see the last chapter of the section "The embryonic nephrogenic program depends on GDNF").

6. The following statement referencing the proportional differences in glomerular density should be revised: "This shows that despite the early deceleration of NP self-renewal, a larger proportion of late born nephrons develop in the kidneys facing excess GDNF during organogenesis." At present it is unlikely to be true as Short et al., 2014 showed that over 50% of nephrons form after birth during normal development. "late born nephrons" would encompass that 50% of nephrons born after birth in the control so without measuring the number of nephrons before birth and into adulthood this is still unclear. The number of nephrons would be much lower in hyper/hyper mice.

Response: Please see also our response to the previous comment. The sentence is now rephrased to "This indicates that despite the early deceleration of NP self-renewal and differentiation, the kidneys facing excess GDNF during organogenesis have ongoing postnatal nephrogenesis with a minor increase in the latest born nephrons (Rumballe et al., 2011)."

7. Regarding the retention of some NP cells after cessation- the data in Figure 3 shows a

© 2021. Published by The Company of Biologists under the terms of the Creative Commons Attribution License (https://creativecommons.org/licenses/by/4.0/).

number of examples where a thin layer of NP cells is present in gdnf hyper/hyper kidneys and not controls. While the Gdnf hyper/hyper mice are clearly delayed in their cessation of nephrogenesis, the staining referenced in Fig4C is more in line with an early committing nephron than persistent NP cells at this stage. Rumballe et al., Dev Biol., 2011, report the transition from NP -> early committing nephron at cessation is marked partly by a change in localisation of NP cells, but primarily by the formation of a Collagen IV+ basement membrane around NP clusters, which coincides with morphological evidence of increasing polarity - seen as a rosette of NP nuclei with a gap in the centre (Rumballe et al., Fig 3b and c). These signs of polarisation are evident in Fig. 4C (arrow on the left), the central arrow points to an isolated cell, the right arrow points to a cluster of cells similar to one marked in the control (asterisk in the right of Fig. 4A). This section could perhaps focus on a consistent delay in gdnf hyper/hyper mice compared to controls.

Response: We agree with the reviewer that P6 nephron progenitors resemble more committed than fully undifferentiated progenitors and this is now also stated in the revised manuscript (p. 6 of results section and in fig.4 legend). However, based on our new analysis of how many of nephrogenic niches have nephron progenitors we would still like to highlight that not all progenitors are committed in *Gdnf*$^{hyper/hyper}$ at P4 (Fig. 4 of revised manuscript).
The results are now described as: "Finally, we found that approximately 18% of the NP niches sustained SIX2-positive NPCs at P4, and committed NPCs were present in *Gdnf*$^{hyper/hyper}$ kidneys until P6, while committed NPCs were detected in WT kidneys only until P4 (Fig. 4)."

8. Authors point out how an excess of GDNF can prolong the nephrogenic program and compare this to other models like Lin28/Let7 overexpression which create a much more dramatic phenotype, but can lead to cancer. However, they fail to acknowledge the dramatic abnormalities associated with the Gdnf hyper/hyper mice.

Response: We agree and apologize for not discussing and contextualizing better the branching morphogenesis defect of *Gdnf*$^{hyper/hyper}$ mice that we have published earlier (Li et al., 2019), and which clearly results in hypodysplastic kidneys.
We do recognize that these renal defects in *Gdnf*$^{hyper/hyper}$ mice evoke severe concerns and describe the limitations of our model now in several sections of the revised manuscript, where also the superiority of GDNF-hyper mice over the other models is now substantially toned down.

9. The authors comment on the phenotype of mice with a single Gdnf hyper allele, stating that this single allele is insufficient to maintain nephron progenitor cells however they only show data from postnatal day 5 (supp fig 5 E,F), which past the point that NP cells are maintained in the gdnf hyper/hyper line.

Response: We apologize for the confusion here. The SIX2 staining to detect nephron progenitors is performed at P4 and P6 stages of wild type and *Gdnf*$^{hyper/hyper}$ kidneys (Fig. 4) The point we want to make with *Gdnf*$^{wt/hyper}$ kidneys stained with SIX2 at P5 that similar to wild type kidneys, they do not show any SIX2 positivity (nor in undifferentiated or committed nephron progenitors). Therefore we do conclude that GDNF increase by single allele 3'UTR disruption is not sufficient to maintain nephron progenitors in postnatal kidneys.
10. Figure 6: are new nephrons formed at P7 or are these the same nephrons that formed at P4? Are these nephrons stalled at an early stage?
Response: Unfortunately we are unable to comprehensively answer to these questions. We do think that based on the persistent presence of nephron progenitors until P4, new nephrons could be still forming in P7 *Gdnf*$^{hyper/hyper}$ kidneys but genetic experiments to verify this are not possible within the timeframe of the revision.
11. The paper focuses on nephron progenitor maintenance and extension of nephrogenesis but NP cells are only visualised early in kidney development (E11.5, 14.5) and at postnatal day 3. It would be useful to know whether this population remains scarce, or has a resurgence between E15.5-P2.
Response: We apologize for the confusing presentation. We show SIX2 and PAX2 staining of nephron progenitors at E11.5, E12.5 (cultured kidneys), E14.5, P0, P3, P4 and P6 (Figs. 1, 3, 4 and 8; S1). Nephron precursors are visualized by different markers at E14.5, E16.5, P0, P3-P7 and P12 (Figs. 2., 5. and 6.; S4)
We included here also additional staining of SIX2 at E16.5 (Fig. SIX2-positive nephron progenitors below). Based on this and the results presented in the manuscript it is in our opinion safe to say

© 2021. Published by The Company of Biologists under the terms of the Creative Commons Attribution License (https://creativecommons.org/licenses/by/4.0/).

that nephron progenitors do not resurgence at any developmental stage in *Gdnf*$^{hyper/hyper}$ kidneys.

We have removed unpublished data provided for the referees in confidence.

12. Comment- Figure 3 D seems to show a strong upregulation of Pax2 in the ureteric tip compared to controls.
13.
Response: Thank you for this notion! This is very interesting and we will address GDNF regulation of Pax2 and other transcription factors in our next study.

Reviewer 3 *Advance Summary and Potential Significance to Field:*

In "Postnatal prolongation of mammalian nephrogenesis by excess fetal GDNF" Hao and colleagues describe the nephron progenitor phenotype of Gdnf hyper mice during the first week of postnatal development.

The authors describe that "excess Gdnf, expands the nephrogenic program beyond its normal cessation by maintaining NP cells and nephrogenesis in postnatal mouse kidneys"

There is an ongoing effort to understand how nephron progenitors can be expanded in vitro for experimental purposes and potential regenerative therapeutic ends, and with a growing number of premature births surviving, it is important to recognize the impact of premature cessation of nephrogenesis and the impact this has on nephron endowment and ultimately quality of life and health. Further, there is an inherent interest in understanding the biology underpinning nephron progenitor growth, expansion and differentiation.

The significance of this manuscript rests on its finding that higher expression of Gdnf can result in a delayed end to nephrogenesis. The delay is from approximately day 4 to day 7 of postnatal development. The debate of whether nephron progenitors commit to differentiate during this period due to an intrinsic or extrinsic clock, or a change in environment, is still on going.

The authors show the following:
1. in Gdnf-hyper mice NPCs persist longer.
2. Fgf9/Fgf20 deficient mice do not display this (argument being hypoplasia is therefore not cause of NPC survival in Gdnf-hyper mice)
3. The Gdnf-hyper mice phenotype can be modified on a Wnt11 het background.

As a reviewer, what I am missing is the obvious connection between these experiments, and some form of cohesive view of a gene regulatory circuit/pathway being modified. How is the biology of NPCs altered on the hyper or hyper/Wnt11 backgrounds? Without these, it is not clear that this warrants publication in Development at this time as the advance remains primarily descriptive.

Response: We thank the reviewer for acknowledging the importance of our results and pointing out the missing characterization of gene regulatory circuits of nephron progenitors in the presence of excess GDNF. We appreciate valuable comments and have now addressed these concerns substantially in the revised manuscript (edits and new results written in red).

Please see our responses to the specific comments below.

Comments for the Author:
1. The authors build up an argument that initial NPC endowment is similar in wildtype and Gdnf- hyper kidneys but this relies on data in Figure 1/S1 and it is not clear how NPCs are quantified, is it per area, per kidney, per tip?

Response: We apologize for incomplete description of the nephron quantification method in our original manuscript. The ureteric bud tips are inherently taken into account in our method, which counts nephron progenitors in their niches = around each ureteric bud tip. This is now described in more detail in the Methods section of the revised manuscript.

© 2021. Published by The Company of Biologists under the terms of the Creative Commons Attribution License (https://creativecommons.org/licenses/by/4.0/).

2. The authors do state the analyses are done on E12.5 kidneys, but if the Gdnf-hyper kidneys have altered branching morphogenesis and tip numbers, are they taking this into account? The authors quantify NPC amounts at E12.5 and argue that the start-point are similar for wt and hyper kidneys. However, in Figure 1A-B there is a clear difference at E11.5 (which is not quantified), and similarly, there is a striking difference at E14.5 (also not quantified) which begs the question of whether the E12.5 data is indeed accurate. Better descriptions of the quantification and the caveats of the quantification method are needed.
The authors also quantify PHH3+ NPCs in wildtype and hyper kidneys as well as in cultured kidneys exposed to Gdnf and show a consistent decrease, concomitant with reduced nephrogenesis. Again, more detailed descriptions of how this was performed and the caveats (as based on the other Gdnf phenotypes exhibited) are needed.

Response: We agree with the critique and have now included both new data and deeper Methods section. Please see also our answer to the first comment.

In addition to quantification of the nephron progenitor numbers at E12.5 in the original manuscript, we have now additionally quantified them at E11.5 and E14.5 kidneys. In the revised ms data description is added on page 5 as: "*Quantification of NPs at E11.5 revealed an initial increase in NPC numbers, which was transiently normalized at E12.5 but severely decreased at E14.5 Gdnf$^{hyper/hyper}$ kidneys (Fig. 1G-H, S1E-F)*".

Furthermore, comparison of the nephron progenitor numbers in *Gdnf*$^{hyper/hyper}$ and *Gdnf*$^{hyper/hyper}$; *Wnt11*$^{+/-}$ at E14.5 and P0 revealed that removal of one *Wnt11* allele resulted in 1,4 and 1,5 higher average of nephron progenitors/niche, respectively (Fig. 8 of revised manuscript).

We have also quantified how many of nephrogenic niches have nephron progenitors in wild type and *Gdnf*$^{hyper/hyper}$ kidneys at P3-P6 (Fig3-4, S1H). This revealed that while in wild types at P3 only 10% of the UB tips (niches) are capped with NPCs (Fig. 3A, 48 tips analyzed in 2 kidneys), 59% of analyzed niches in *Gdnf*$^{hyper/hyper}$ have NPs at P3 (Fig. 3B, 107 tips analyzed in 2 kidneys). At P4 and P5 NPCs were no longer detected in wild type niches (Fig. 4A-B, 315 and 10 tips analyzed, respectively). Of note, it was really difficult to find calbindin-positive epithelium resembling tip morphology in the cortex of P6 wild type kidneys, which substantially restricted the number of tips available for analyzes. In P4 *Gdnf*$^{hyper/hyper}$ kidneys NPCs were present in 18% of the analyzed niches while at P6 only committed NPCs were detected (Fig. 4C- D, 245 and 16 tips analyzed, respectively).

3. The authors describe cortical to cortical-to-total glomerular density as a measurement where wildtype and hyper kidneys differ. This raises the question, are all renal corpuscles not typically positioned in the cortex? The is a fairly sharp boundary between the Outer Medulla and the Cortex where renal corpuscle, S1, and S2 are within the Cortex. Could the authors elaborate on the meaning of this wording and how the analyses were performed?

Response: We agree with the critique and apologize for incomplete description of the glomeruli quantification method in our original manuscript. As the reviewer acknowledges, there is a clear boundary between cortical and outer medulla regions in the kidney, and these are present in late embryonic and early postnatal kidneys. We first measured the kidney area from where the glomeruli were counted in each section by Fiji program. The total number of glomeruli and number of glomeruli in the cortex were then counted separately. The average measured area, overall and cortical glomeruli numbers were then calculated for each sample. Average glomerular density was calculated by dividing the average glomerular number by the average of measured area. The average cortical glomerular density was calculated by dividing the average cortical glomerular number by the average of measured total area. This does not give real cortical glomerular density but rather indicates the amount of glomeruli located in the cortex.

In the revised manuscript we have now included the description of our calculation to the Methods section of revised manuscript (p.18 new, text in red). We have also re-labelled what we called average cortical glomerular density to average cortical glomerular density within the total area in the revised manuscript to more faithfully present our calculations (Table 1.). Finally, we describe the glomerular density results in a manner that gives the reader more thorough understanding of

© 2021. Published by The Company of Biologists under the terms of the Creative Commons Attribution License (https://creativecommons.org/licenses/by/4.0/).

effects that the fetal excess of GDNF has on renal differentiation in general and especially on glomerular density, which is not improved to the level of wild type kidneys (p. 5 of the revised manuscript). It now runs:

"*Glomerular density assessment further supported this and demonstrated lower overall densities in hypodysplastic Gdnf$^{hyper/hyper}$ kidneys than in wild type (WT) kidneys (Fig S1G). Interestingly, analysis of the cortical glomeruli within the total measured area and especially cortical-to-total glomeruli ratios revealed slightly higher than WT ratios in Gdnf$^{hyper/hyper}$ (Table 1; 17% in Gdnf$^{hyper/hyper}$, 12% in WT). This indicates that despite the early deceleration of NP self-renewal and differentiation, the kidneys facing excess GDNF during organogenesis have ongoing postnatal nephrogenesis with minor increase in the latest born nephrons (Rumballe et al., 2011).*"
"However, our analysis failed to detect SIX2- and/or PAX2-positive cells in the NP niche of Fgf9;20 deficient kidneys (Supplemental figure 2). This together with published data (O'Brien, 2018; Urbach et al., 2014; Yermalovich et al., 2019) suggests that prolonged nephrogenesis derives from increased GDNF, not from renal hypoplasia." While this is possible, it could also be due to renal hypoplasia. I would argue a negative outcome here i.e. non-detection of Six2 NPCs in Fgf9/Fgf20 deficient kidneys, does not suggest a positive conclusion. Renal hypoplasia can occur due to many conflicting situations. All the authors can conclude is that the phenotype in Fgf9/Fgf20 deficient mice is not the same as in the Gdnf-hyper kidneys. Without the underlying mechanism it is hard to make further arguments.

Response: Renal hypoplasia as a possible cause of continued postnatal nephrogenesis is a very important aspect of our study. It was originally addressed in FGF9;20 mutant kidneys, and thorough literature search. We agree that although neither of these indicate that small size (=hypoplasia) per se would be able to maintain nephron progenitors longer than in "normal sized" kidney, they do not comprehensively exclude that possibility.
Recently several new studies demonstrate wide variability in healthy kidney size and timing of nephrogenesis cessation (Bennett et al., 2020; Bonsib, 2020; Charlton et al., 2020; Kuure and Sariola, 2020; Ryan et al., 2018; Sutherland et al., 2011). None of these shows or suggests correlation between reduced kidney size and persistence of nephrogenesis, which should manifest as small kidneys where glomerular density is higher than in normal sized kidneys.
In the revised manuscript we have now additionally analyzed the postnatal presence of nephron progenitors in a model where renal hypoplasia is caused by impaired ureteric bud branching (similarly as in *Gdnf*$^{hyper/hyper}$ mice), namely *Hoxb7Cre; Mek1;Mek2* mice. Deletion of all four alleles results in very severe renal hypodysplasia and newborn lethality (Ihermann-Hella et al., 2014) but inactivation of ¾ alleles is compatible with life as kidneys show only mildly reduced size. Again, no signs of prolonged nephrogenesis could be detected (Fig. S3), strongly supporting the view that renal hypoplasia as such is not enough to maintain postnatal nephrogenesis.
Based on these and other results we present in the revised manuscript we think that increased fetal GDNF expression, not the small kidney size it causes, has a major impact on nephron progenitors. To acknowledge the imperfection of our experimentation and in agreement of "the negative result does not suggest a positive conclusion" in the results section we now write: "Also published data demonstrates that renal hypoplasia alone is unable to support postnatal NP maintenance (Kuure and Sariola, 2020), which further suggests active role for GDNF but cannot completely exclude the possibility that increased fetal amount of other growth factor signals, which may or may not reduce kidney size, will not have the same effect." (p. 6-7).
"Removal of one functional Wnt11 allele in Gdnfhyper/hyper background (Gdnfhyper/hyper;Wnt1+/-) did not improve postnatal kidney size from that in Gdnfhyper/hyper pups (Supplemental figure 7A-E). However, removal of Wnt11 allele in Gdnfhyper/hyper individuals enhanced SIX2-positive NP population and improved nephrogenesis, as evidenced by increased PAX2-positivity in the cortical differentiation zone of Gdnfhyper/hyper;Wnt1+/- kidneys (Figure 8, compare also to Figure 1F). Thus, this result suggests that WNT11 mediates GDNF's regulatory functions to NP cells."
There is a typo in the above sentence but more importantly this is not supported by clear quantification of Six2+ progenitor populations.

Response: Here we refer to our response to the reviewer's comment 2: We have now quantified nephron progenitors at all stages from which there is data presented in our manuscript. This includes comparison of the nephron progenitor numbers in *Gdnf*$^{hyper/hyper}$ and *Gdnf*$^{hyper/hyper}$; *Wnt11*$^{+/-}$ at E14.5 and P0. This validates our original conclusion that removal of one *Wnt11* allele in

© 2021. Published by The Company of Biologists under the terms of the Creative Commons Attribution License (https://creativecommons.org/licenses/by/4.0/).

Gdnf$^{hyper/hyper}$ background results in enhanced nephron progenitor maintenance (1,4 and 1,5 higher average of nephron progenitors/niche, respectively) and supports also improved nephrogenesis (Fig. 8 of revised manuscript).

Further, knowing that modifications to Gdnf signalling alters branching morphogenesis, is it not plausible that a higher level of Gdnf protein results in expanded tips, with consequent larger Wnt11 expression domains? The Gdnfhyper/hyper; Wnt11+/- could therefore just have restored the tip size? The authors describe this phenotype in S7 (red arrows). Following on from that the authors describe "cyst in glomerular tuft (black arrow)" in S7D-E. The glomerular tuft refers to the capillaries that form the glomerulus. To me it looks like these cysts are made from an expansion of the Bowman's space.

Response: We agree with the reviewer that the expanded ureteric bud in *Gdnf*$^{hyper/hyper}$ kidneys, caused by the excess expression of GDNF, likely contributes to the increased expression of not only *Wnt11* but other transcriptional targets of GDNF as well as *Wnt9b*. Indeed, in the revised manuscript we now show that increased *Wnt11* and *-9b* are accompanied by significantly increased expression of canonical WNT target *Axin2* at E14.5 kidneys (Fig. S7A in revised manuscript). Of note, excess canonical WNT signaling is previously shown to negatively impact nephron progenitor maintenance (Dapkunas et al., 2019; Kiefer et al., 2012). Furthermore, our new experiments show that chemical inhibition of WNT signaling in the presence of excess GDNF alleviates extended ureteric bud tip morphology (Fig. S8). Of note, WNT inhibition by IWR1 and IWP2 (data not shown) disrupts nephron progenitor coherence, which likely derives from the imbalance the chemicals cause to progenitor autonomous WNT signaling.

In postnatal kidneys the nephron progenitor specific canonical WNT targets (Karner et al., 2011) *Cited1* and *Uncx4.1* together with *R-spondin1*, a known Wnt signaling agonist, which together *R-spondin3* is essential for the nephron progenitor maintenance and differentiation (de Lau et al., 2014; Vidal et al., 2020), showed increased expression in *Gdnf*$^{hyper/hyper}$ kidneys (Fig. S7B). These new results together with our data in Fig. 7 suggest that increased GDNF levels augment several bud- derived GDNF/Ret targets, which not alone but in combination with each other and together with increased canonical WNT signaling are inhibitory to nephron progenitor proliferation and differentiation in embryonic kidneys. In postnatal kidneys, GDNF expression gradually decreases and generates permissive environment for normal nephrogenesis. The results also suggest that GDNF may act as a previously unrecognized master regulator of ureteric bud-derived signals that function to control events in the nephrogenic niche.

We thank the reviewer for pointing out incorrect nomenclature used in previous legend for the fig. S7D-E. We have now changed the labeling of what we previously called glomerular tuft to Bowman's space in the figure legend of fig. S9 in the revised manuscript.

"Gdnf, expressed by the nephron progenitor population, is lost from wild type kidneys by P2 (www.gudmap.org) while its mRNA and protein were still present in Gdnfhyper/hyper kidneys as late as P7 (Figure 7A-D)."

Given the importance of understanding the dynamics of Gdnf expression during postnatal days of development, it is surprising to me that the authors did not perform a time-course of in situ hybridization experiments to pinpoint the exact differences between wildtype and hyper kidneys.

Response: To state what the reviewer cites above we indeed have analyzed *Gdnf* expression by in situ hybridization through postnatal (and developmental) stages (P0-P6) to compare its localization in wild type and *Gdnf*$^{hyper/hyper}$ kidneys (figure below). As shown for P4 in the main figure 7A-B of the revised manuscript, this reveals increased expression without major changes in the transcript localization pattern, reflecting well rest of the result of the figure (Fig. 7C-D). It is worth mentioning that qRT-PCR is much more sensitive method than in situ hybridization and thus can detect traces of expression in tissues where the latter method fails to show transcripts.

We have removed unpublished data provided for the referees in confidence.

I was under the impression that mouse genes are written as follows Gdnf while their protein are Gdnf. This contrasts to human nomenclature GDNF/GDNF.

Response: We have carefully double-checked all gene and protein names in the manuscript, and can confirm that we do adhere to official guidelines on nomenclature rules as laid out by http://www.informatics.jax.org/mgihome/nomen/. In essence, mouse gene symbols begin with an

© 2021. Published by The Company of Biologists under the terms of the Creative Commons Attribution License (https://creativecommons.org/licenses/by/4.0/).

uppercase letter followed by all lowercase letters and are italicized in published articles (*Gdnf*). Protein designations follow the same rules as gene symbols, with the distinctions that they use all uppercase letters and are not italicized (SIX2).

Supplemental figure 1 lacks texts describing S1F.

.

Response: We apologize for the missing legend. In the revised manuscript Fig. S1F, is designated as Table 1 and the legend is included.

References:

Bennett, K. M., Baldelomar, E. J., Morozov, D., Chevalier, R. L. and Charlton, J. R. (2020). New imaging tools to measure nephron number in vivo: opportunities for developmental nephrology. *J Dev Orig Health Dis*, 1-5.
Bonsib, S. M. (2020). Renal Hypoplasia, From Grossly Insufficient to Not Quite Enough: Consideration for Expanded Concepts Based Upon the Author's Perspective With Historical Review. *Adv Anat Pathol* **27**, 311-330.
Charlton, J. R., Baldelomar, E. J., Hyatt, D. M. and Bennett, K. M. (2020). Nephron number and its determinants: a 2020 update. *Pediatr Nephrol*.
Dapkunas, A., Rantanen, V., Gui, Y., Lalowski, M., Sainio, K., Kuure, S. and Sariola, H. (2019). Simple 3D culture of dissociated kidney mesenchyme mimics nephron progenitor niche and facilitates nephrogenesis Wnt-independently. *Sci Rep* **9**, 13433.
de Lau, W., Peng, W. C., Gros, P. and Clevers, H. (2014). The R-spondin/Lgr5/Rnf43 module: regulator of Wnt signal strength. *Gene Dev* **28**, 305-316.
Ihermann-Hella, A., Lume, M., Miinalainen, I. J., Pirttiniemi, A., Gui, Y., Peranen, J., Charron, J., Saarma, M., Costantini, F. and Kuure, S. (2014). Mitogen-activated protein kinase (MAPK) pathway regulates branching by remodeling epithelial cell adhesion. *PLoS Genet* **10**, e1004193.
Karner, C. M., Das, A., Ma, Z., Self, M., Chen, C., Lum, L., Oliver, G. and Carroll, T. J. (2011). Canonical Wnt9b signaling balances progenitor cell expansion and differentiation during kidney development. *Development* **138**, 1247-1257.
Kiefer, S. M., Robbins, L. and Rauchman, M. (2012). Conditional expression of Wnt9b in Six2-positive cells disrupts stomach and kidney function. *PLoS One* **7**, e43098.
Kuure, S., Sainio, K., Vuolteenaho, R., Ilves, M., Wartiovaara, K., Immonen, T., Kvist, J., Vainio, S. and Sariola, H. (2005). Crosstalk between Jagged1 and GDNF/Ret/GFRalpha1 signalling regulates ureteric budding and branching. *Mech Dev* **122**, 765-780.
Kuure, S. and Sariola, H. (2020). Mouse Models of Congenital Kidney Anomalies. *Adv Exp Med Biol* **1236**, 109-136.
Li, H., Jakobson, M., Ola, R., Gui, Y., Kumar, A., Sipila, P., Sariola, H., Kuure, S. and Andressoo, J. O. (2019).
Development of the urogenital system is regulated via the 3'UTR of GDNF. *Sci Rep* **9**, 5302.
Lu, B. C., Cebrian, C., Chi, X., Kuure, S., Kuo, R., Bates, C. M., Arber, S., Hassell, J., MacNeil, L., Hoshi, M., et al. (2009). Etv4 and Etv5 are required downstream of GDNF and Ret for kidney branching morphogenesis. *Nat Genet* **41**, 1295-1302.
Menshykau, D., Michos, O., Lang, C., Conrad, L., McMahon, A. P. and Iber, D. (2019). Image-based modeling of kidney branching morphogenesis reveals GDNF-RET based Turing-type mechanism and pattern-modulating WNT11 feedback. *Nat Commun* **10**, 239.
Ola, R., Jakobson, M., Kvist, J., Perala, N., Kuure, S., Braunewell, K. H., Bridgewater, D., Rosenblum, N. D., Chilov, D., Immonen, T., et al. (2011). The GDNF Target Vsnl1 Marks the Ureteric Tip. *J Am Soc Nephrol* **22**, 274-284.
Rumballe, B. A., Georgas, K. M., Combes, A. N., Ju, A. L., Gilbert, T. and Little, M. H. (2011). Nephron formation adopts a novel spatial topology at cessation of nephrogenesis. *Dev Biol* **360**, 110-122.
Ryan, D., Sutherland, M. R., Flores, T. J., Kent, A. L., Dahlstrom, J. E., Puelles, V. G., Bertram, J. F., McMahon, A. P., Little, M. H., Moore, L., et al. (2018). Development of the Human Fetal Kidney from Mid to Late Gestation in Male and Female Infants. *EBioMedicine* **27**, 275-283.
Sainio, K., Suvanto, P., Davies, J., Wartiovaara, J., Wartiovaara, K., Saarma, M., Arumae, U., Meng, X., Lindahl, M., Pachnis, V., et al. (1997). Glial-cell-line-derived neurotrophic factor is required for bud initiation from ureteric epithelium. *Development* **124**, 4077-4087.
**Sutherland, M. R., Gubhaju, L., Moore, L., Kent, A. L., Dahlstrom, J. E., Horne, R. S., Hoy, W. E., Bertram, J.
F. and Black, M. J.** (2011). Accelerated maturation and abnormal morphology in the preterm

© 2021. Published by The Company of Biologists under the terms of the Creative Commons Attribution License (https://creativecommons.org/licenses/by/4.0/).

neonatal kidney. *J Am Soc Nephrol* **22**, 1365-1374.
Vidal, V. P., Jian-Motamedi, F., Rekima, S., Gregoire, E. P., Szenker-Ravi, E., Leushacke, M., Reversade, B., Chaboissier, M. C. and Schedl, A. (2020). R-spondin signalling is essential for the maintenance and differentiation of mouse nephron progenitors. *Elife* **9**.

Second decision letter

MS ID#: DEVELOP/2020/197475

MS TITLE: Postnatal prolongation of mammalian nephrogenesis by excess fetal GDNF

AUTHORS: Hao Li, Kristen Kurtzeborn, Jussi Kupari, Yujuan Gui, Edward Siefker, Benson Lu, Kart Matlik, Soophie Olfat, Ana R Montano-Rodriguez, Sung-Ho Huh, Franklin D Costantini, Jaan-Olle Andressoo, and Satu Kuure
ARTICLE TYPE: Research Article

I am happy to tell you that your manuscript has been accepted for publication in Development, pending our standard ethics checks.

Reviewer 1

Advance summary and potential significance to field

The findings in this manuscript are novel in the research field of developmental nephrology, and may also be useful for medical strategies to regulate congenital nephron endowment.

Comments for the author

The authors have adequately addressed most of my concerns.

Reviewer 2

Advance summary and potential significance to field

This work presents a new observation that nephron progenitor cells can be maintained past the point that they would usually differentiate by increased levels of gdnf. This finding and the authors work to explain the mechanistic basis of this outcome is a novel contribution to our understanding of nephrogenesis and how it might be extended to increase nephron number.

Comments for the author

The authors have addressed the points raised by this reviewer. The revised manuscript presents the results in a more balanced way, and in a fuller context of the authors prior work and related work in the field. The additional quantification and clarification provided has substantially improved how the work comes across.
The new inhibitor studies in Figure S8 are interesting. If images of higher magnification or resolution are available please include them in any future version of this manuscript to allow readers to see more detail on how nephron progenitor cell localisation has been affected by IWR1.

© 2021. Published by The Company of Biologists under the terms of the Creative Commons Attribution License (https://creativecommons.org/licenses/by/4.0/).
